# FUNDC1-dependent mitochondria-associated endoplasmic reticulum membranes are involved in angiogenesis and neoangiogenesis

**DOI:** 10.1038/s41467-021-22771-3

**Published:** 2021-05-10

**Authors:** Cheng Wang, Xiaoyan Dai, Shengnan Wu, Wenjing Xu, Ping Song, Kai Huang, Ming-Hui Zou

**Affiliations:** 1 Center for Molecular and Translational Medicine, Georgia State University, Atlanta, Georgia USA; 2 Clinic Center of Human Gene Research, Union Hospital, Tongji Medical College, Huazhong University of Science and Technology, Wuhan, China

**Keywords:** Endoplasmic reticulum, Mitochondria, Angiogenesis, Tumour angiogenesis

## Abstract

FUN14 domain-containing protein 1 (FUNDC1) is an integral mitochondrial outer-membrane protein, and mediates the formation of mitochondria-associated endoplasmic reticulum membranes (MAMs). This study aims to determine the contributions of FUNDC1-mediated MAMs to angiogenesis in vitro and in vivo. In cultured endothelial cells, VEGF significantly increases the formation of MAMs and MAM-related proteins, including FUNDC1. Endothelial cell-specific deletion of FUNDC1, which disrupts MAM formation in endothelial cells, lowers VEGFR2 expression and reduces tube formation, spheroid-sprouting, and functional blood vessel formation in vitro and in vivo. Conversely, increased MAM formation using MAM linkers mimics the effects of VEGF and promotes endothelial angiogenesis. Mechanistically, increased MAMs formation led to increased levels of Ca^2+^ in cytosol, promoted the phosphorylation of serum response factor (SRF) and enhanced the binding of SRF to VEGFR2 promoter, resulting in increased VEGFR2 production, with consequent angiogenesis. Moreover, blocking FUNDC1-related MAM formation with a cell-penetrating inhibitory peptide significantly suppresses the expressions of downstream angiogenic genes and inhibits tumor angiogenesis. We conclude that decreased MAMs formation by silencing FUNDC1 can inhibit angiogenesis by decreasing VEGFR2 expression, and targeting FUNDC1-dependent MAMs might be a promising approach for treating human disorders characterized by defective angiogenesis.

## Abstract (web summary)

Mitochondria-associated ER membranes (MAMs) are involved in the regulation of many cellular functions. Here, the authors report that FUNDC1-dependent mitochondria-associated endoplasmic reticulum membranes play an essential role in the process of angiogenesis and neoangiogenesis.

## Introduction

Angiogenesis is crucial for the development and repair of tissues, and thereby has an important role in embryonic development, tissue growth, and wound healing. Aberrant neovascularization, however, is an essential pathogenic mechanism of tumor progression and metastasis^1–3^. It is a two-edged sword, and a better understanding of the angiogenic process can provide an appropriate approach for intervention. One of the main angiogenic signaling mechanisms that mediate both developmental and pathological angiogenesis involves vascular endothelial growth factor (VEGF) and VEGF receptors^4,5^. While intracellular signaling pathways resulting from VEGF stimulation have been extensively studied, the signal transduction pathways culminating in the biological consequences of VEGF/VEGFR signaling are only partially understood.

The mitochondria and endoplasmic reticulum (ER) are key structures in the regulation of many cellular functions. They are interconnected and share structural and functional interactions essential for cellular homeostasis. Mitochondria form close physical contacts with a specialized ER domain known as the mitochondria-associated ER-membrane (MAM)^6,7^. Alterations in MAM signaling have pleiotropic effects on multiple diseases, including inflammation, immune responses, Alzheimer’s disease, Parkinson’s disease, lysosomal storage diseases, and dysfunction of lipid and glucose metabolism^8,9^. Additionally, increasing evidences highlight that ER–mitochondria contacts are linked to a variety of cellular processes ranging from lipid metabolism, Ca^2+^ signaling, intracellular trafficking of mitochondria and ER to cell survival, energy metabolism, protein folding, and autophagy^10–14^. MAMs could mediate the shuttling of phosphatidylcholine between ER and mitochondria, which is necessary for the maintenance of mitochondrial tubular morphology and therefore for mitochondrial functions^15^. Mitochondria are prone to taking up large amounts of Ca^2+^ due to their close juxtaposition with the ER. Constitutive Ca^2+^ transfer by MAMs to mitochondria is essential to maintain cell bioenergetics in normal cells and its absence induces bioenergetic stress and activates prosurvival macroautophagy^16^. The transmembrane protein inositol 1,4,5-trisphosphate receptor (IP3R) is the main factor responsible for Ca^2+^ release from the ER. In endothelial cells, mitochondria can associate directly with IP3Rs to form MAMs^17^. IP3Rs at MAM regions regulate the highly efficient transfer of Ca^2+^ between the ER and mitochondria, thereby controlling fundamental processes involved in mitochondrial oxidative metabolism and energy production. Likewise, prolonged mitochondrial Ca^2+^ overload compromises mitochondrial function by causing a transient collapse of the mitochondrial membrane potential, leading to necrosis or apoptosis^18,19^.

FUN14 domain-containing 1 (FUNDC1) is a mammalian mitophagy receptor that interacts with and recruits LC3 to mitochondria for mitophagy^20^. Recently, several studies have shown that FUNDC1 also has an important role in mitochondrial function by controlling the integrity and function of MAMs^21,22^. In the MAMs, FUNDC1 can promote mitochondrial fission by directly interacting with dynamin-related protein 1 (DRP1) at ER–mitochondria contact sites^22^. As a novel MAM protein, FUNDC1 controls a variety of physiological and pathological processes, such as mitochondrial dynamics, Ca^2+^ dyshomeostasis, and autophagy^23^. Our group recently reported that FUNDC1 can modulate ER Ca^2+^ release into mitochondria through interacting with IP_3_R2, initiate aberrant mitochondrial fission, and lead to cardiac dysfunction and heart failure^21^. More interestingly, diabetes could initial FUNDC1-mediated MAMs formation in cardiomyocytes, causing mitochondrial dysfunction, and lead to cardiomyopathy^24^. However, the role of FUNDC1 and FUNDC1-related MAMs in vascular biology remains largely unknown.

In this study, our results show that diminishing FUNDC1-dependent MAM formation in endothelial cells inhibits angiogenesis, whereas increasing MAMs promote angiogenesis. Mechanistically, defective FUNDC1-related MAM formation disrupts intracellular Ca^2+^ homeostasis, thereby reducing VEGFR2 levels and inhibiting angiogenesis.

## Results

### VEGF increases FUNDC1-related MAM formation in endothelial cells

**Fig. 1:**
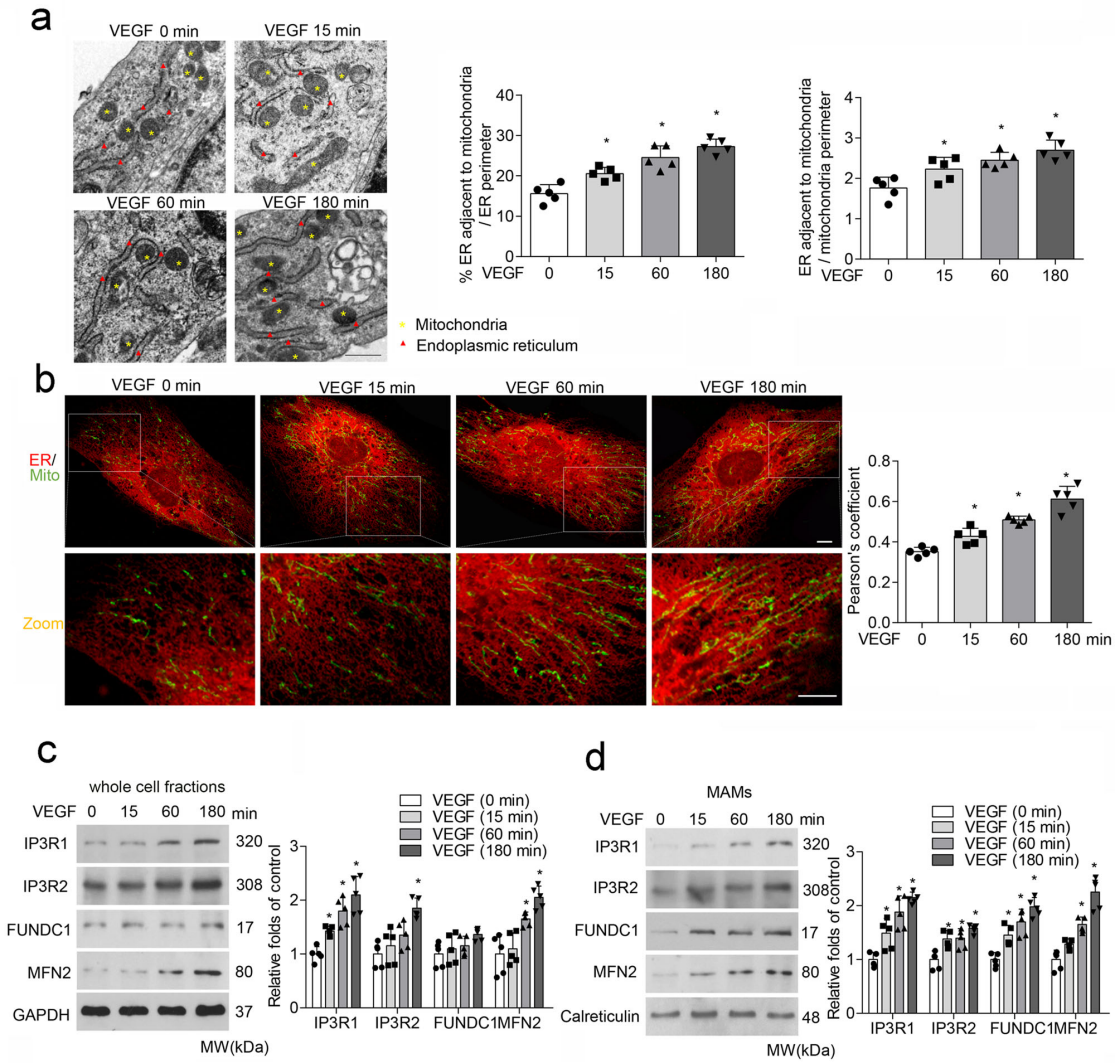
VEGF treatment increases MAMs formation in endothelial cells. Human umbilical vein endothelial cells (HUVECs) were treated with vascular endothelial growth factor (VEGF; 30 ng/mL) for 0, 15, 60, and 180 min. **a** Representative transmission electron microscopy images of ER and mitochondrial morphology. Bar graph illustrating quantitation of ER length adjacent to mitochondria normalized by total ER length and by the mitochondrial perimeter. (*n* = 5 independent experiments). Scale bars, 500 nm. **b** Association between the endoplasmic reticulum (ER) and mitochondria (Mito) was analyzed by confocal microscopy. Representative confocal images are shown. Bar graph showing quantification of ER–mitochondria contacts using the Pearson’s coefficient. Scale bars, 10 µm (*n* = 5 independent experiments). **c** Western blot analysis of mitochondria-associated endoplasmic reticulum membrane (MAM)-related proteins in VEGF-treated HUVECs. Relative expression was quantified by densitometric analysis of the western blot assay (*n* = 5 independent experiments). **d** Western blot analysis of MAM-related proteins in endothelial MAM fractions prepared from VEGF-treated HUVECs. Relative expression was quantified by densitometric analysis of the western blot assay (*n* = 5 independent experiments). Statistical significance was assessed using two-tailed *t*-tests for two groups and one-way ANOVA with post hoc multiple comparisons test for comparing multiple groups. *p < 0.05 vs VEGF (0 min). All values are mean ± S.D.

We first assessed MAM formation by analyzing the apposition of ER and mitochondria in endothelial cells. As illustrated by transmission electron microscope (TEM) images in Fig. 1a, the association of ER and mitochondria was markedly increased by VEGF stimulation in a time-dependent manner. Similarly, immunofluorescent staining and Pearson’s correlation coefficient analysis revealed a time-dependent increase in co-localization of ER and mitochondria in VEGF-treated cells (Fig. 1b). Next, we examined MAM-associated protein levels in whole-cell fractions of VEGF-treated endothelial cells. The results showed that VEGF significantly increased the expression of proteins involved in MAM formation and Ca^2+^ transport, such as IP3R1, IP3R2, and MFN2 in a time-dependent manner (Fig. 1c). To further analyze MAM protein levels in the MAM fraction, we performed Percoll-based subcellular fractionation to collect enriched fractions of MAMs from endothelial cells, and observed an increased accumulation of IP3R1, IP3R2, FUNDC1, and MFN2 in mitochondria–ER contacts in the VEGF-treated groups (Fig. 1d). Taken together, these findings indicate that VEGF treatment increases co-localization of ER and mitochondria and expressions of FUNDC1-associated MAM-related proteins in endothelial cells.

### Disrupting MAMs by FUNDC1 silencing inhibits angiogenesis

**Fig. 2:**
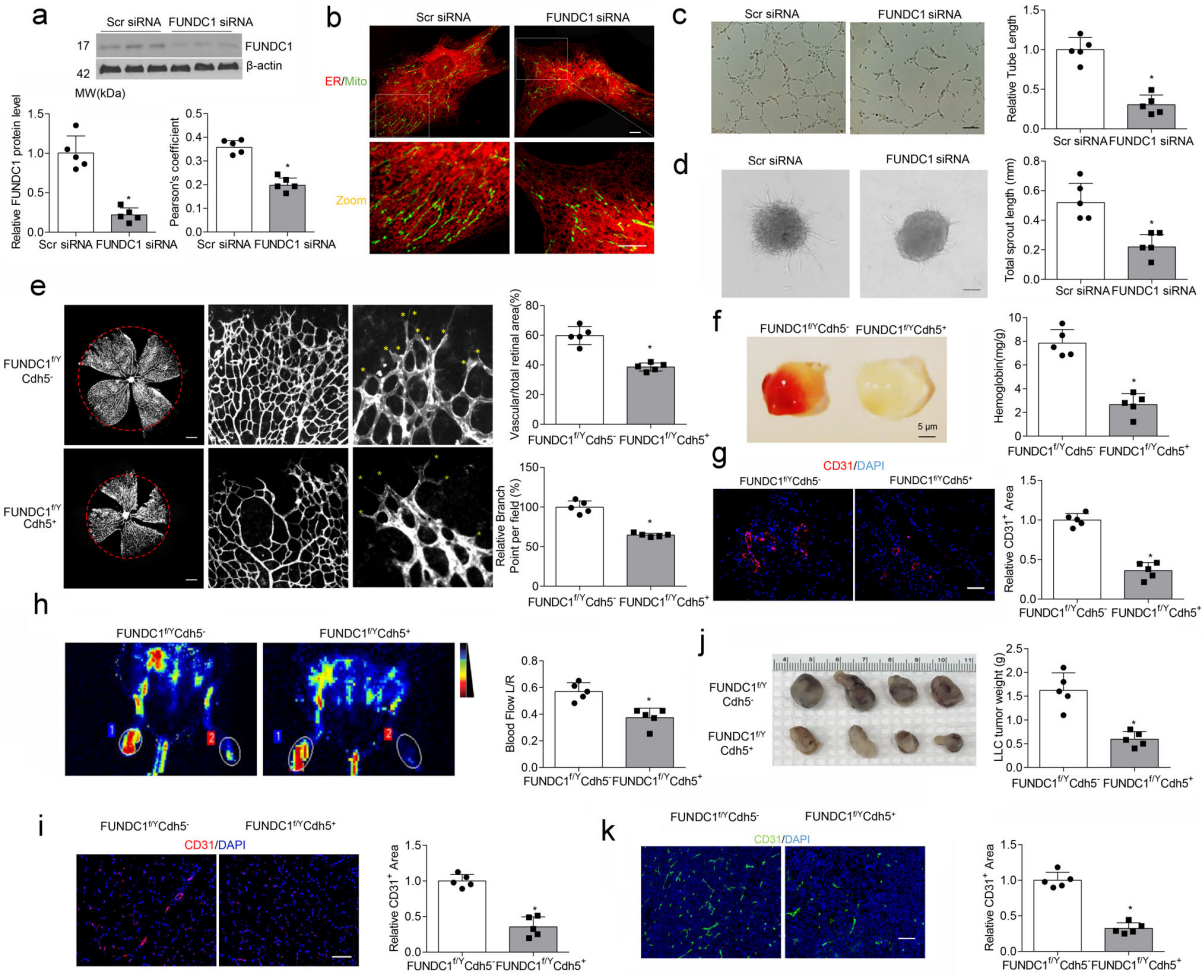
MAM disruption by FUNDC1 deficiency reduces angiogenesis in vitro and in vivo. **a** HUVECs were transfected with scrambled (scr) siRNA or FUN14 domain-containing 1 (FUNDC1) siRNA for 24 h. The protein levels of FUNDC1 were determined by western blot assays (*n* = 5 independent experiments). **b** HUVECs were transfected with Scr siRNA or FUNDC1 siRNA for 24 h. Representative confocal images are shown. Quantification of endoplasmic reticulum (ER)–mitochondria contacts using Pearson’s coefficient. (*n* = 5 independent experiments). Scale bars, 10 µm. **c** HUVECs transfected with Scr siRNA or FUNDC1 siRNA were plated onto matrigel. Representative images of tube formation are shown (left). Tube length/field in 10 random microscopic fields per group was measured by NIH ImageJ and statistically analyzed (*n* = 5 independent experiments) (right). Scale bar, 100 µm. **d** HUVECs were transfected with Scr siRNA or FUNDC1 siRNA for 24 h. Three-dimensional spheroids were generated (left), and representative images of spheroid-sprouting were analyzed (*n* = 5 independent experiments) (right). Scale bar, 100 µm. **e** Representative confocal images of a P5 retina lobe stained with IsoB4 showing reduced number of sprouts (yellow stars) at the vascular front of *FUNDC1*^*f/Y*^*Cdh5*^*+*^ retinas compared with *FUNDC1*^*f/Y*^*Cdh5*^−^ retinas. (*n* = 5 mice/group). Scale bars, 500 mm. **f** Matrigel plugs implanted into *FUNDC1*^*f/Y*^*Cdh5*^−^ and *FUNDC1*^*f/Y*^*Cdh5*^*+*^ mice for 10 days. Quantification of hemoglobin (Hb) extracted from matrigel plugs from *FUNDC1*^*f/Y*^*Cdh5*^−^ and *FUNDC1*^*f/Y*^*Cdh5*^*+*^ mice (*n* = 5 mice/group). **g** CD31 immunostaining of matrigel plugs implanted into *FUNDC1*^*f/Y*^*Cdh5*^−^ and *FUNDC1*^*f/Y*^*Cdh5*^*+*^ mice for 10 days (left). Quantification of the relative CD31-positive area (fold-over control) (right). (*n* = 5 mice/group). Scale bar, 100 µm. **h** Representative images showing blood flow reperfusion assessed by Doppler laser ultrasound on day 10 after ischemic injury. (*n* = 5 mice/group). The ratio of ischemic/non-ischemic perfusion was quantitatively analyzed. **i** Representative images showing anti-CD31 immunostaining in gastrocnemius muscle on day 10 after femoral artery ligation. (*n* = 5 mice/group). Scale bar, 100 µm. **j**, **k** Tumors following subcutaneous injection of Lewis lung carcinoma (LLC) tumor cells into the abdomen of *FUNDC1*^*f/Y*^*Cdh5*^−^ and *FUNDC1*^*f/Y*^*Cdh5*^*+*^ mice. After 28 days, tumors were harvested and quantified. Scale bar, 100 µm. **j** Representative images of tumors (*n* = 5 mice/group). **k** Immunostaining of LLC tumor sections and quantification of relative CD31-positive areas. (*n* = 5 mice/group). Scale bar, 100 µm. Statistical significance was assessed using two-tailed t-tests to compare two groups. *p < 0.05 vs Scr siRNA or *Fundc1*^*f/Y*^*Cdh5*^−^. All values are mean ± S.D.

FUNDC1, a highly conserved protein that modulates communication between ER and mitochondria and MAM formation^22^, co-localized with both mitochondria and ER in HUVECs (Supplementary Fig. 1), and FUNDC1-deleted endothelial cells exhibited reduced co-localization between ER and mitochondria, compared with control siRNA-transfected cells (Fig. 2a, b). We subsequently disrupted MAMs formation by silencing *FUNDC1* expression to evaluate the effects of MAMs on angiogenesis in vitro and in vivo. In the tube formation assay, *FUNDC1* ablation significantly reduced tube length to an average of 30% of the values in control endothelial cells (Fig. 2c). In the spheroid-sprouting assay, a decrease in total sprout length was observed in *FUNDC1*-deficient endothelial cells (Fig. 2d).

In vivo, we generated EC-specific *FUNDC1* knockout mice (*FUNDC1*^*f/Y*^*Cdh5*^*+*^) to determine the contributions of FUNDC1 and MAMs in endothelial function. The knockout efficiency of the *FUNDC1* gene was verified, and reduced ER–mitochondria contacts were determined by electron microscopy assay (Supplementary Fig. 3). Whole-mount preparations of retinas from *FUNDC1*^*f/Y*^*Cdh5*^*+*^ mice on postnatal day 5 (P5) demonstrated a delayed expansion of the vascular plexus to the periphery after MAM disruption, compared with controls, as evidenced by a decrease in vascular branching and reduced vessel coverage (Fig. 2e). In addition, matrigels mixed with VEGF were injected subcutaneously into 6-week-old *FUNDC1*^*f/Y*^*Cdh5*^*−*^ and *FUNDC1*^*f/Y*^*Cdh5*^*+*^ mice and then removed out on day 10. Angiogenesis was delayed in the matrigel plugs from *FUNDC1*^*f/Y*^*Cdh5*^*+*^ mice, as evidenced by less Hb content (Fig. 2f) and reduced CD31-positive area in the plugs noted on confocal images (Fig. 2g). In the hindlimb ischemic model, blood flow was significantly attenuated in *FUNDC1*^*f/Y*^*Cdh5*^*+*^ mice after ischemic injury, compared with *FUNDC1*^*f/Y*^*Cdh5*^−^ mice (Fig. 2h). Capillary density (assessed by anti-CD31 immunostaining) in skeletal muscle on day 10 after ischemic injury was an average of 0.65-fold lower in *FUNDC1*^*f/Y*^*Cdh5*^*+*^ mice than that in *FUNDC1*^*f/Y*^*Cdh5*^−^ mice (Fig. 2i), suggesting delayed angiogenesis after MAM disruption.

As angiogenesis is essential for tumor growth and metastasis, controlling tumor-associated angiogenesis is a promising approach for limiting cancer progression^25^. We next tested whether MAMs could regulate tumor angiogenesis. After injecting LLC cells into the abdomens of 6-week-old *FUNDC1*^*f/Y*^*Cdh5*^−^ and *FUNDC1*^*f/Y*^*Cdh5*^*+*^ mice, we evaluated tumor growth and microvessel density. Tumor size was approximately 65% lower in *FUNDC1*^*f/Y*^*Cdh5*^*+*^ mice than that in *FUNDC1*^*f/Y*^*Cdh5*^−^ mice (Fig. 2j). Peritumoral microvasculature was analyzed by vessel density via immunostaining for CD31 expression in LLC-derived tumors. Endothelial FUNDC1 deficiency markedly suppressed CD31 expression in tumors (Fig. 2k). Therefore, loss of endothelial MAMs by FUNDC1 deletion impairs angiogenesis.

### Induced MAMs formation by a synthetic linker accelerate angiogenesis

**Fig. 3:**
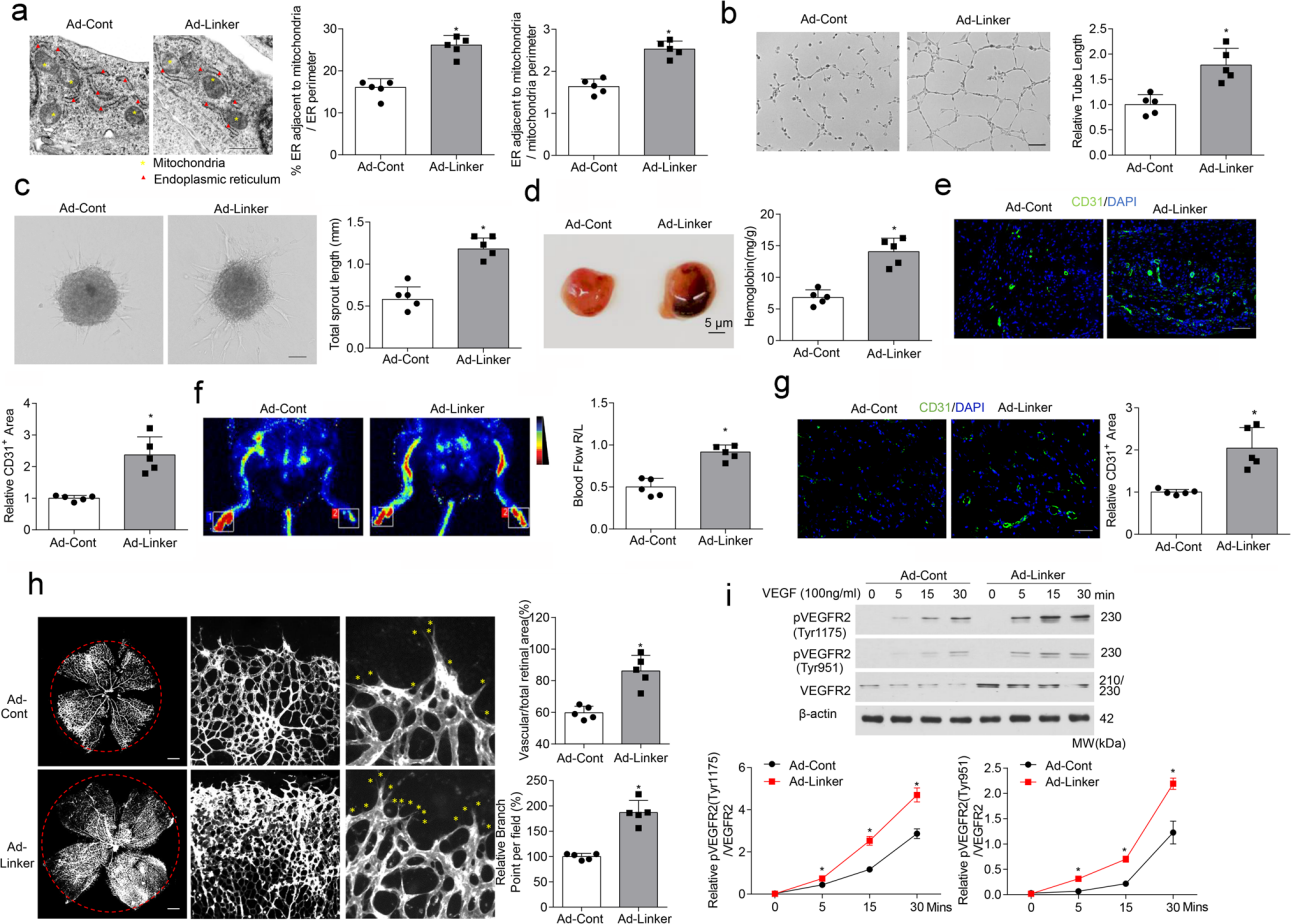
MAM induction enhances angiogenesis in vitro and in vivo. HUVECs were infected for 24 h with adenovirus encoding control (Ad-Cont) or mitochondria–endoplasmic reticulum (ER) linker (Ad-Linker) (*n* = 5 independent experiments). **a** Representative transmission electron microscopy images of ER and mitochondrial morphology. Bar graph illustrating quantitation of ER length adjacent to mitochondria normalized by total ER length and by mitochondrial perimeter (*n* = 5 independent experiments). **b** Representative images of tube formation. (*n* = 5 independent experiments). Scale bar, 100 µm. Tube length/field in 10 random microscopic fields per group was measured by NIH ImageJ and statistically analyzed. **c** Representative images of spheroid-sprouting. (*n* = 5 independent experiments). Scale bar, 100 µm. Sprout length in 10 random microscopic fields per group was measured by NIH ImageJ and statistically analyzed. **d** Matrigel containing vascular endothelial growth factor (VEGF) was injected subcutaneously into 6-week-old mice that been injected intravenously with adenoviruses coding control (Ad-Cont) or mitochondria–ER linker (Ad-Linker) 24 h before. After 10 days, matrigel plugs were removed for analysis of new vessel formation by histological examination and hemoglobin (Hb) assay (*n* = 5 mice/group). Bar graph illustrating quantification of Hb extracted from matrigel plugs of different groups. **e** Anti-CD31 immunostaining of matrigel plugs from different groups. (*n* = 5 mice/group). Scale bar, 100 µm. Bar graph showing quantification of relative CD31-positive area (fold of control). **f** Representative images showing blood flow reperfusion monitored by laser Doppler ultrasound on day 10 after ischemic injury. The ratio of ischemic/non-ischemic perfusion was quantitatively analyzed (*n* = 5 mice/group). **g** Representative images showing CD31 immunostaining in the gastrocnemius muscle on day 10 after artery ligation. (*n* = 5 mice/group). Scale bar, 100 µm. **h** Representative confocal images of a P5 retina lobe stained with IsoB4 showing a higher number of sprouts (yellow stars) at the vascular front of Ad-Linker infected retinas, compared with Ad-Cont infected retinas. (*n* = 5 mice/group). Scale bars, 500 mm. **i** Western blot analysis of phosphorylation of VEGFR2 at Try 1175 and Try 951 (*n* = 4 independent experiments). Statistical significance was assessed using two-tailed *t*-tests to compare two groups. *p < 0.05 vs Ad-Cont. All values are mean ± S.D.

Then we used the mitochondria–ER linker to increase MAMs formation in HUVECs and assessed its function on angiogenesis. Co-localization of ER and mitochondria in endothelial cells was indeed greater in Ad-linker group than that in Ad-Cont (Fig. 3a). We first performed in vitro tube formation and spheroid-sprouting assays. MAMs over-formation by the linker markedly increased tube length and sprout length, compared with the Ad-Cont group (Fig. 3b, c). The synthetic linker was next transduced into mice in vivo. In Matrigel plug assays, the linker markedly increased angiogenesis, as shown by more Hb accumulation (Fig. 3d) and larger CD31-positive area in plugs from Ad-linker-treated mice (Fig. 3e). In the ischemic experiments, more blood flow in Ad-linker group was observed after ischemic injury (Fig. 3f), and the relative capillary density in skeletal muscles was an average of 2.04-fold higher than that in control mice (Fig. 3g). We also determined the role of MAMs in regulating postnatal angiogenesis in vivo. Adenoviruses encoding ER–mitochondrial linker or Cont were injected into the vitreous of C57BL/6J wild-type mice. Quantification of whole-mount retinas at P5 showed an increase in vascular area, blood vessel branching, and radial outgrowth, all indicating that MAMs enhance angiogenic formation (Fig. 3h).

Consistent with these data, we also overexpressed FUNDC1 to mediate higher MAMs formation, which showed that FUNDC1 overexpression significantly increased co-localization of ER and mitochondria (Supplementary Fig. 2a). Ectopic FUNDC1 expression promoted endothelial vessel sprouting and tube formation (Supplementary Fig. 2b, c). In matrigel plug assays, FUNDC1 overexpression accelerated neovascularization, as shown by increased Hb content in the plugs (Supplementary Fig. 2d). Together, our findings indicate that accelerating MAMs formation by FUNDC1 overexpression also enhances endothelial angiogenesis.

MAMs overexpression was proved to promote angiogenesis, which mimicked the effects of VEGF^26^, so we went on exploring the downstream signaling pathway of VEGF under MAMs overexpression in endothelial cells. Phosphorylation of VEGFR2 at Tyr1175 and Tyr951 sites was all increased with Ad-linker infection (Fig. 3i). These results suggest that MAMs can possibly stimulate the VEGFR2 signaling pathway and subsequently enhance angiogenesis.

### VEGFR2 mediates the angiogenic effects of MAMs

**Fig. 4:**
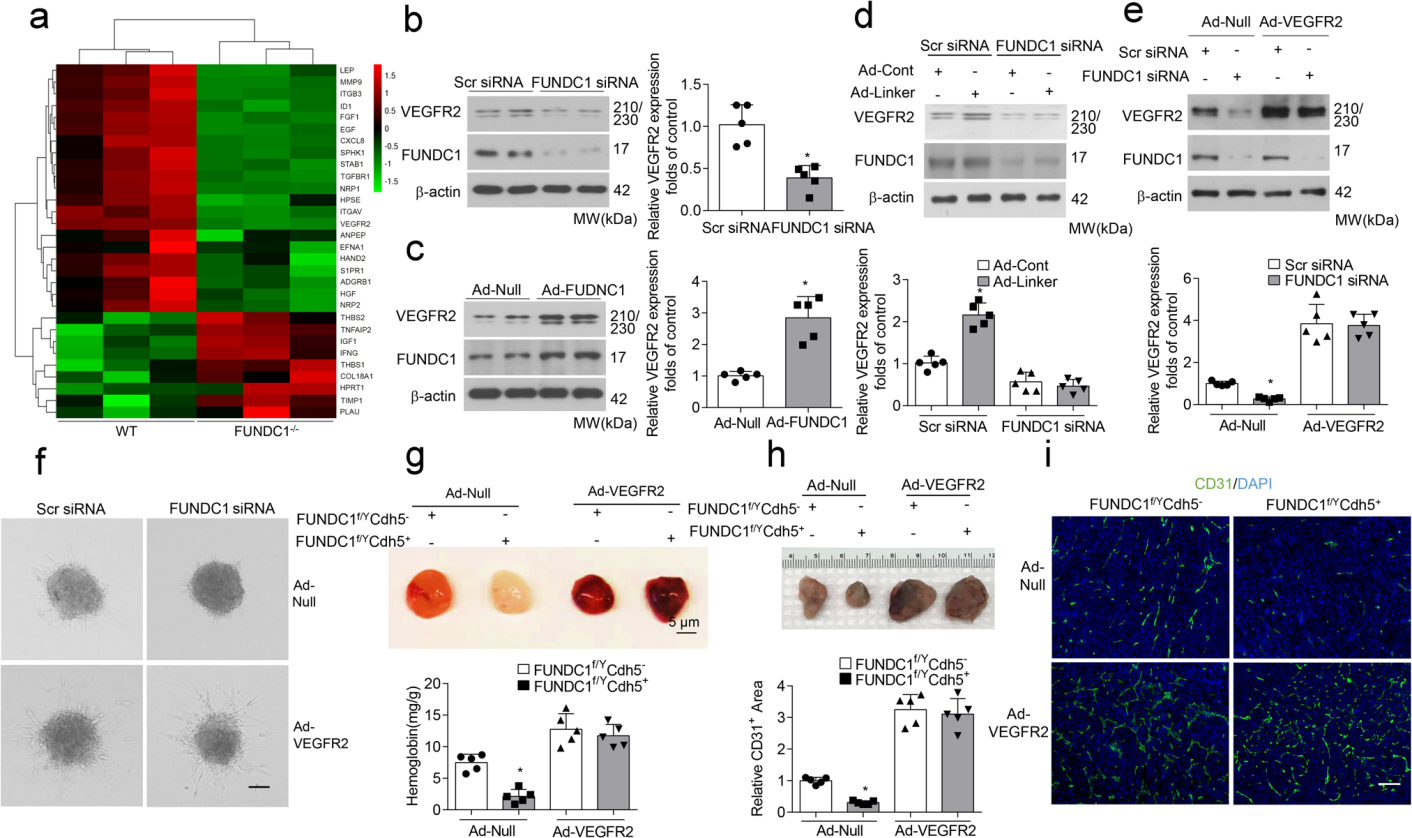
MAM-mediated angiogenesis is dependent on VEGFR2. **a** Angiogenesis PCR array for *FUNDC1*^*f/Y*^*Cdh5*^−^ and *FUNDC1*^*f/Y*^*Cdh5*^*+*^ endothelial cells. The heat map depicts relative expression values for the 30 discrepant genes (*n* = 3 biologically independent samples per group). **b** HUVECs were transfected with Scr siRNA or FUNDC1 siRNA for 24 h, the protein level of vascular endothelial growth factor receptor 2 (VEGFR2) were determined by western blot assays (*n* = 5 independent experiments). **c** HUVECs were infected with Ad-null or Ad-FUNDC1 for 24 h, and protein level of VEGFR2 were determined by RT-qPCR and western blot assays (*n* = 5 independent experiments). **d** HUVECs were transfected with Scr siRNA or FUNDC1 siRNA for 24 h and then infected with adenovirus encoding control (Ad-Cont) or mitochondrial–endoplasmic reticulum (ER) linker (Ad-Linker) for another 24 h (*n* = 5 biologically independent samples per group). Expression of VEGFR2 and FUNDC1 was determined by western blot assay. **e** HUVECs were transfected with Scr siRNA or FUNDC1 siRNA for 24 h and then infected with adenovirus encoding null or VEGFR2 for another 24 h (*n* = 5 independent experiments). Expression of VEGFR2 and FUNDC1 was determined by western blot assay. **f** Representative images of spheroid-sprouting are shown. Sprout length in ten random microscopic fields per group were measured by NIH ImageJ and statistically analyzed. (*n* = 5 independent experiments). Scale bar, 100 µm. **g** Matrigel containing vascular endothelial growth factor (VEGF) was injected subcutaneously into *FUNDC1*^*f/Y*^*Cdh5*^−^ and *FUNDC1*^*f/Y*^*Cdh5*^*+*^ mice that had received intravenous injections of VEGFR2 adenoviruses 24 h before. After 10 days, matrigel plugs were removed for analysis of new vessel formation by histological and Hb assays (*n* = 5 mice/group) (top). Quantification of Hb extracted from Matrigel plugs from *FUNDC1*^*f/Y*^*Cdh5*^−^ and *FUNDC1*^*f/Y*^*Cdh5*^*+*^ mice (*n* = 5 mice/group) (bottom). **h** Established LLC tumors approximately 90 mm^3^ in size were treated with either intravenous injection of recombinant VEGFR2 adenoviruses or Ad-Null as control *(n* = 5 mice/group). Scale bar, 100 µm. After 28 days, the tumors were harvested and quantified. Representative images of tumors were shown. **i** Immunostaining of LLC tumor sections and quantification of relative CD31-positive area. (*n* = 5 mice/group). Scale bar, 100 µm. Statistical significance was assessed using two-tailed *t*-tests for two groups and using one-way ANOVA with post hoc multiple comparisons test for multiple groups. *p < 0.05 vs Scr siRNA; Ad-Null or *FUNDC1*^*f/Y*^*Cdh5*^−^+Ad-Null. All values are mean ± S.D.

We next analyzed angiogenesis-related gene expression under FUNDC1 manipulation. An angiogenesis PCR array screened that the expression of growth factor and receptor genes was markedly downregulated in FUNDC1-deficient endothelial cells, especially for the gene *VEGFR2*, the main receptor for VEGF-induced angiogenesis (Fig. 4a). The mRNA and protein levels of VEGFR2 in endothelial cells were then verified using real-time PCR and western blot assay, respectively. Results showed that FUNDC1 deficiency decreased *VEGFR2* expression, whereas FUNDC1 overexpression increased *VEGFR2* expression (Fig. 4b, c and Supplementary Fig. 4). We also examined VEGFR2 levels in wild-type and FUNDC1-deficient endothelial cells, which were infected with the mitochondria–ER Linker. FUNDC1 depletion blocked the upregulation of VEGFR2 in HUVECs by induced ER–mitochondrial contacts (Fig. 4d). These results suggest that the function of FUNDC1-related MAMs in angiogenesis involves VEGFR2 signaling pathways.

Therefore, it is rational to suspect that VEGFR2 possibly mediated the effects of MAMs. To evaluate this hypothesis, HUVECs, pre-transfected with FUNDC1 siRNA, were infected with adenovirus encoding null or VEGFR2 (Fig. 4e). In spheroid-sprouting assays, ectopic expression of VEGFR2 consistently reversed the decreased sprout length and number of branching points due to FUNDC1 deletion (Fig. 4f). In vivo*, FUNDC1*^*f/Y*^*Cdh5*^*−*^ and *FUNDC1*^*f/Y*^*Cdh5*^*+*^ mice were transduced with adenoviruses containing VEGFR2 or null. In the matrigel plug assay, overexpression of VEGFR2 also reversed the decreased Hb content and CD31-positive area in the plugs (Fig. 4g). Furthermore, in the LLC-derived tumor model, ectopic VEGFR2 expression antagonized the decreased CD31-positive vessel density occurring with FUNDC1 deficiency, thus enhancing tumor growth and reversing the reduced tumor size (Fig. 4h, i). Additionally, VEGFR2 knockdown abolished the enhanced sprout length induced by the MAMs linker (Supplementary Fig. 5). Collectively, these data suggest that VEGFR2 mediates the angiogenic effects of FUNDC1-related MAMs.

### FUNDC1-related MAMs upregulate VEGFR2 expression in a Ca^2+^/SRF-dependent transcriptional manner

**Fig. 5:**
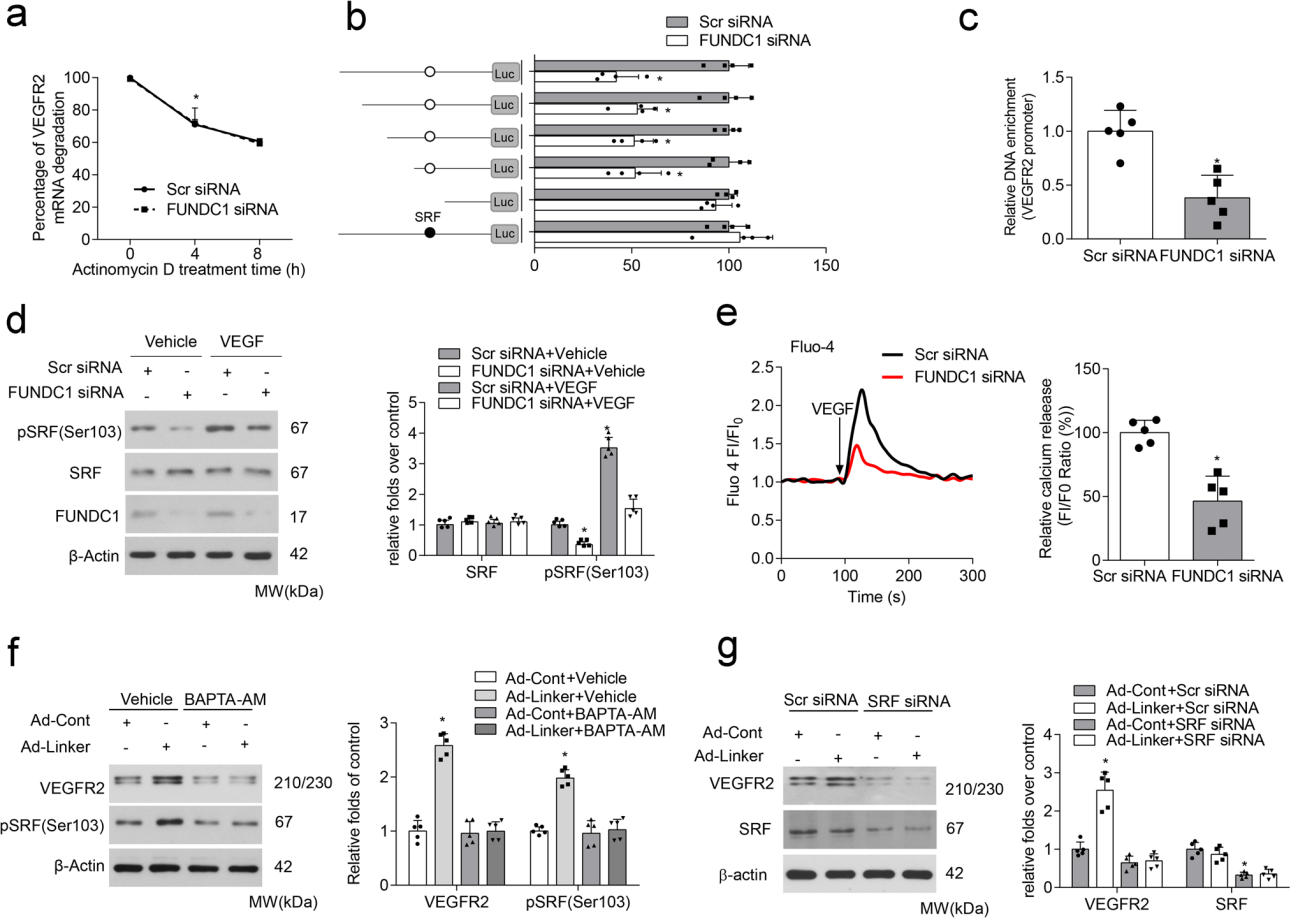
SRF mediates the positive regulation of MAMs on VEGFR2 expression. **a** HUVECs were pre-transfected with or without FUNDC1 siRNA for 24 h, then treated with actinomycin D for different periods of time. RT-qPCR assay analysis of vascular endothelial growth factor receptor 2 (VEGFR2) mRNA levels (*n* = 5 independent experiments). **b** HUVECs were pre-transfected with or without FUNDC1 siRNA for 24 h, then transfected with various VEGFR2 promoter truncation constructs or a mutant (serum response factor [SRF] binding site) construct for 24 h. After this, luciferase activity was measured (*n* = 5 independent experiments). **c** Chromatin immunoprecipitation assays using an anti-SRF antibody to amplify VEGFR2 promoter in HUVECs transfected with or without FUNDC1 siRNA (*n* = 5 independent experiments). **d** HUVECs were pre-transfected with or without FUNDC1 siRNA for 24 h and then treated with VEGF for 30 min. Expression of SRF and phosphorylated SRF at Ser103 (pSRF) was determined by western blot assay (*n* = 5 independent experiments). **e** Cytosolic Ca^2+^ levels, as indicated by the fluorescent probe Fluo-4, AM (*n* = 5 independent experiments). HUVECs were transfected with Scr siRNA or FUNDC1 siRNA for 24 h and then exposed to VEGF (20 ng/mL) for the indicated times. **f** HUVECs were infected with adenovirus encoding control (Ad-Cont) or mitochondrial–endoplasmic reticulum (ER) linker (Ad-Linker) for 24 h and then treated with BAPTA-AM (10 µM) or control dimethylsulfoxide (vehicle) for 30 min. Levels of SRF and pSRF (Ser103) were detected by western blot assay (*n* = 5 independent experiments). **g** HUVECs were pre-transfected with or without SRF siRNA for 24 h, then infected with adenovirus encoding control or mitochondria–ER linker for 24 h. VEGFR2 protein expression was detected by western blot assay (*n* = 5 independent experiments). Statistical significance was assessed using two-tailed *t*-tests for two groups and one-way ANOVA with post hoc multiple comparisons test for multiple groups. *p < 0.05 vs Scr siRNA; Scr siRNA+VEGF; Ad-Cont+Vehicle or Ad-Cont+Scr siRNA. All values are mean ± S.D.

Considering that FUNDC1 regulated VEGFR2 expression at both mRNA and protein levels, we wondered whether this regulation occurred mainly in mRNA synthesis or mRNA decay. Actinomycin D was used to inhibit endogenous mRNA transcription, and the half-life (*t*_1/2_) of *VEGFR2* mRNA was determined. Results showed that FUNDC1 did not affect *VEGFR2* mRNA degradation (Fig. 5a). Next, we constructed a series of luciferase reporter plasmids containing different lengths of the VEGFR2 promoter, including pGL3 (control), pGL3-VEGFR2-40 (−40 to +121 bp), pGL3-VEGFR2-125 (−125 to +121 bp), pGL3-VEGFR2-280 (−280 to +121 bp), pGL3-VEGFR2-420 (−420 to +121 bp), and pGL3-VEGFR2-570 (−570 to +121 bp), and performed luciferase activity assays to explore the possibility of transcriptional regulation. Interestingly, FUNDC1 deficiency markedly suppressed the luciferase activity of four plasmids (pGL3-VEGFR2-125, pGL3-VEGFR2-280, pGL3-VEGFR2-420, and pGL3-VEGFR2-570), but not the activity of pGL3 (control) or pGL3-VEGFR2-40, indicating that the region essential for VEGFR2 regulation is located between −125 and −40 bp. We screened one consensus SRF-binding element candidate in the region. SRF was previously confirmed to be a Ca^2+^-sensitive transcriptional factor that facilitates gene transcription^27,28^. To confirm the importance of this region, we deleted the putative binding site around −50 bp in pGL-VEGFR2-570 to generate pGL-VEGFR2-570-Δ. The repressed luciferase activity of this plasmid was sharply abolished, regardless of co-transfection with FUNDC1 siRNA, demonstrating that the SRF-binding site around −50 bp was crucial for FUNDC1 regulation of VEGFR2 promoter activity (Fig. 5b). We also tested the binding activity of SRF to this VEGFR2 promoter region under FUNDC1 deficiency. ChIP assays showed that SRF bound directly to this region, but the binding affinity was markedly decreased in FUNDC1-deficient HUVECs (Fig. 5c). Collectively, these results indicate that FUNDC1 promotes SRF-binding to the VEGFR2 promoter and accelerates VEGFR2 transcription, thus leading to increased expression of VEGFR2.

SRF is reported to have a critical role in angiogenesis^29,30^, and its phosphorylation is linked to the induction of tissue factor promoter; this led us to explore whether FUNDC1 affected SRF phosphorylation to initiate VEGFR2 transactivation. Western blot analysis showed that phosphorylation of SRF (at Ser103), but not the total level of SRF, was significantly suppressed by FUNDC1 deficiency in HUVECs after VEGF treatment (Fig. 5d). But how FUNDC1-dependent MAMs regulated SRF phosphorylation remained misty. FUNDC1 has a critical role in intracellular Ca^2+^ homeostasis^21^, and disrupted MAMs formation by silencing FUNDC1 expression indeed resulted in lower levels of intracellular Ca^2+^ in HUVECs (Fig. 5e), so we wondered whether Ca^2+^ acted as a messenger to construct a bridge for communication between MAMs and nuclear SRF. Ca^2+^ levels in ECs were pharmacologically downregulated with BAPTA-AM, which reduces Ca^2+^ levels, under MAMs overexpression. We also verified that BAPTA-AM ablated Linker-induced SRF phosphorylation and the downstream VEGFR2 expression (Fig. 5f), strongly suggesting the role of Ca^2+^ in the regulation of FUNDC1 on SRF. More importantly, silencing of SRF abolished the increase in VEGFR2 expression induced by adenovirus mitochondria–ER linker (Fig. 5g), Taken together, MAMs upregulate VEGFR2 expression in a Ca^2+^/SRF-dependent transcriptional manner.

### A peptide that blocks the FUNDC1–IP3R1 interaction suppresses angiogenesis

**Fig. 6:**
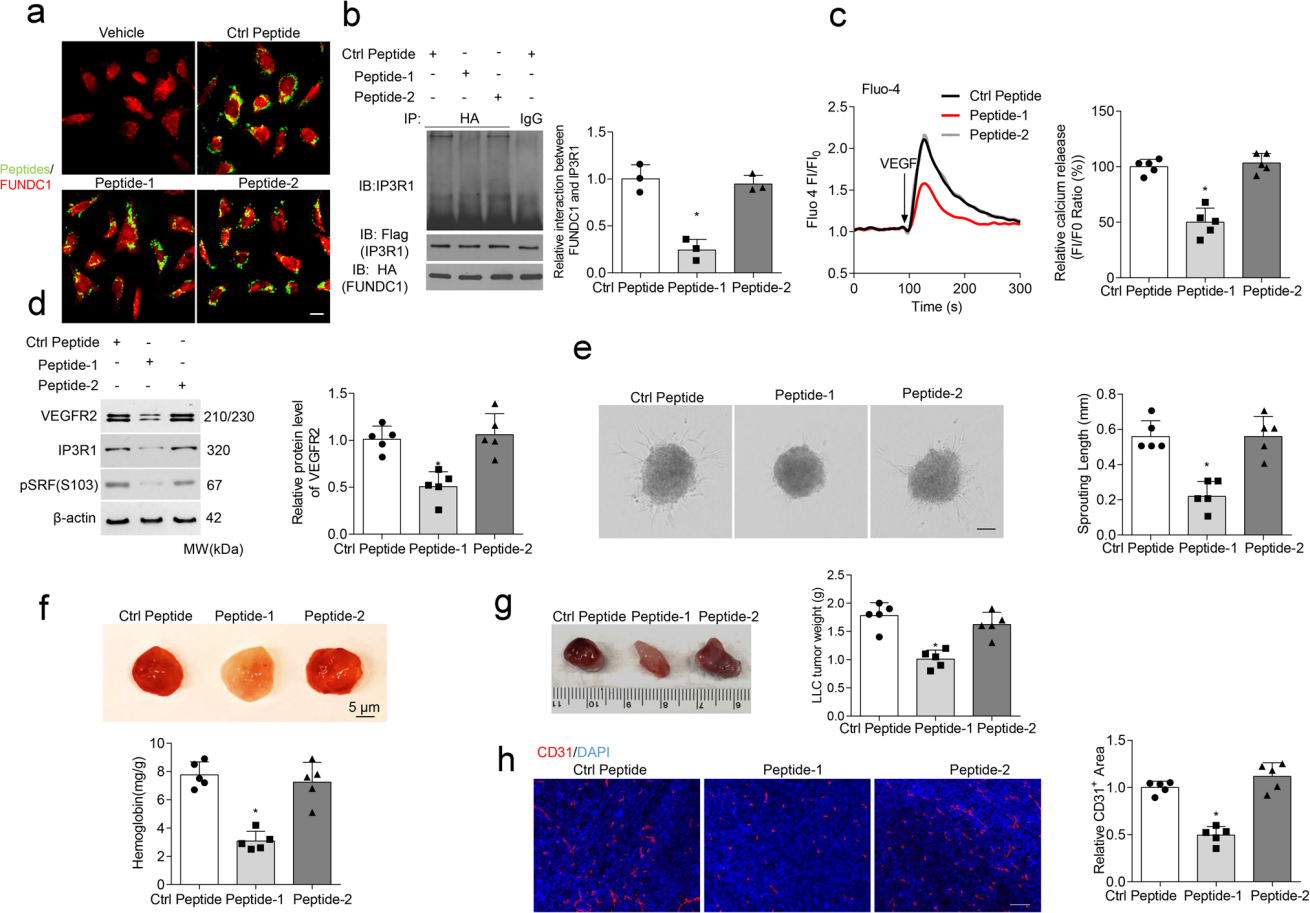
A specific therapeutic peptide blocks the FUNDC1–IP3R1 interaction and angiogenesis. **a** Transduction efficiency of peptides 1, 2, and a control peptide in Human umbilical vein endothelial cells (HUVECs) (*n* = 5 independent experiments). **b** HUVECs were treated with different peptides (20 μM) for 24 h, then subjected to immunoprecipitation with antibody against FUNDC1(HA) to quantify the interaction of FUNDC1 and IP3R1 (*n* = 3 independent experiments). **c** HUVECs were treated with different peptides (20 μM) for 24 h, then cytosolic Ca^2+^ was detected using the fluorescent probe Fluo-4, AM (*n* = 5 independent experiments). **d** HUVECs were treated with different peptides. After 24 h, the cell lysates were collected and subjected to western blot assays to detect the expression of VEGFR2, IP3R1, SRF, and phosphorylated SRF at Ser103 (pSRF) (*n* = 5 independent experiments). **e** Three-dimensional spheroids and representative images of spheroid-sprouting were analyzed (*n* = 5 independent experiments). Scale bar, 100 µm. **f** Matrigel containing vascular endothelial growth factor (VEGF) was injected subcutaneously into 6-week-old wild-type mice, then the mice received an intravenous injection of the indicated peptides. After 10 days, matrigel plugs were removed for analysis of new vessel formation by histological and Hb assay (*n* = 5 mice/group). Quantification of Hb extracted from matrigel plugs of different groups. **g** C57BL/6 mice with LLC tumors that were approximately 90 mm^3^ in size were treated with intravenous injections of different peptides (10 mg/kg/3 days). After 28 days, the tumors were harvested and quantified. Representative images of tumors (*n* = 5 mice/group). **h** Immunostaining of LLC tumor sections and quantification of relative CD31-positive area. (*n* = 5 mice/group). Scale bar, 100 µm. Statistical significance was assessed using one-way ANOVA with post hoc multiple comparisons test for multiple groups. *p < 0.05 vs Ctrl peptide. All values are mean ± S.D.

IP3R functions as a crosstalk station between Ca^2+^ signaling on MAMs^31^. FUNDC1 binds to IP3R and regulates Ca^2+^ release from the ER into the mitochondria and cytosol in cardiomyocytes^21^. Then disrupting this interaction might be an alternative. Firstly, we proved that FUNDC1 could directly interact with IP3R1, the major subtype of IP3R in the endothelial cells, and this interaction was enhanced after VEGF treatment (Supplementary Fig. 6a). More importantly, we have constructed different IP3R1 truncations and revealed that the channel pore domain of IP3R1 protein, especially aa2216–2590 was crucial for its binding to FUNDC1 (Supplementary Fig. 6b). According to this result, we designed two cell-penetrating inhibitory peptides containing 12 amino acids (peptide-1, YGRKKRRQRRRGVIIDTFADLRS; peptide-2, YGRKKRRQRRRLALILVYLFSI), which were based on the degree of homology in the conserved interactive region among different IP3R isoforms at aa2216–2590. They are predicted to have beta-turn and/or a-helical structures, rather than random-coil or nonstructured sequences. HUVECs exposed to these peptides exhibited clear peptide aggregation within the cytoplasm and potential co-localization with FUNDC1 (Fig. 6a). Peptide-1, but not peptide-2, abolished the interaction between FUNDC1 and IP3R1 in cells (Fig. 6b), implying that peptide-1 impairs MAM formation (Supplementary Fig. 10). Administration of peptide-1 also reduced VEGF-induced Ca^2+^ levels (Fig. 6c). In addition, peptide-1 could inhibit the levels of VEGFR2, phosphorylated SRF (Ser103), and IP3R1 in HUVECs (Fig. 6d).

Next, we investigated the therapeutic effects of the peptides on angiogenesis both in vitro and in vivo. Administration of peptide-1, compared with control peptide or peptide-2, delayed endothelial spheroid-sprouting (Fig. 6e). Plugs exposed to peptide-1 showed reduced Hb accumulation (Fig. 6f). More interestingly, intravenous injections of peptide-1 led to a statistically significant reduction in volume and weight of subcutaneous xenografts and the CD31-positive vessel density (Fig. 6g, h). Collectively, these results indicate that an inhibitory peptide blocking MAMs formation via FUNDC1 and IP3R1 can suppress angiogenesis in vitro and in vivo.

## Discussion

In the present study, we have demonstrated the crucial role of FUNDC1-dependent MAMs in neovascularization. Inducing MAM formation increased, but diminishing MAMs by specific FUNDC1 deletion inhibited, angiogenesis both in vitro and in vivo. Disruption of FUNDC1-related MAM formation contributed to intracellular Ca^2+^ dyshomeostasis, resulting in decreased levels of pSRF and VEGFR2, and subsequent reduction of VEGF-induced angiogenesis.

Mitochondria and ER have essential roles in cell physiology through the control of multiple signal transduction pathways. ER and mitochondria continuously exchange messages via MAMs^32^. Dysregulation of communication between mitochondria and ER has a crucial role in multiple human diseases^9^. Recent reports have shown that chronic increases in MAM formation result in mitochondrial Ca^2+^ overload, impairing mitochondrial bioenergetic function and increasing reactive oxygen species (ROS) production in the liver of obese mice^33,34^. Furthermore, mitochondria–ER contact sites function as modulators of ROS synthesis, which lead to aging-associated pathologies, such as Alzheimer’s disease, Parkinson’s disease, and amyotrophic lateral sclerosis^35,36^. In recent years, MAMs and mitochondrial dynamics have likewise been recognized as key factors in the pathogenesis of cardiac and vascular diseases. Our lab demonstrated that suppression of FUNDC1-mediated MAM formation in cardiomyocytes caused cardiac dysfunction^21^. Interestingly, the current experiments provided compelling evidence that genetic deletion of FUNDC1-related MAMs could inhibit angiogenesis. Consistently, enhanced MAM formation, through the mitochondria–ER linker or by increasing FUNDC1 expression, promoted angiogenesis. In addition, a therapeutic peptide blocking MAM-related interactions between FUNDC1 and IP3R1 inhibited angiogenesis.

MAMs are rich in specific proteins, such as the calcium channel IP3R, FUNDC1, chaperones such as Sig 1R and calnexin, the mitochondrial-ER tether proteins MFN2 and PACS2, and proteins involved in phospholipid metabolism^37^. Our data showed that expressions of IP3R, FUNDC1, and MFN2 were significantly elevated in MAM fractions isolated from VEGF-treated endothelial cells. The interaction between FUNDC1 and IP3R1 in MAMs mediated the changes in Ca^2+^ levels. IP3R1 knockdown significantly inhibited vascular angiogenesis in vivo (Supplementary Fig. 7a). VEGFR2 expression was also downregulated after IP3R1 deficiency in HUVECs, whereas expressions of VEGF, VEGFR1, and VEGFR3 were nearly unchanged (Supplementary Fig. 7b). Consistently, a recent study found that loss of MFN1 or MFN2 led to diminished endothelial cell migration and differentiation into network structures in response to VEGF-A stimulation^38^. Still now, there is a total lack of evidence that whether other known modulators on the ER-membrane contacts, like GRP75, PACS2, and Sig1R et al. could be involved in the angiogenesis process, even though they could form Ca^2+^-sensitive chaperone complex in the MAMs and influences Ca^2+^ signaling from the ER to the mitochondria^17^, like FUNDC1/IP3R1. Surprisingly, Dr. Hyun-Woo Rhee reported nearly FUNDC2, not FUNDC1, was found in MAMs by the method contact-ID^39^. Thus, it is of interest to further investigate the actual factors in MAMs and their potential involvement in the regulation of angiogenesis in the future. Although our work demonstrated that enhanced MAM formation by the mitochondria–ER linker or increasing FUNDC1 expression promoted angiogenesis in vitro and in vivo, it is still lacking of direct proof to demonstrate that the effect seen is pure angiogenesis and acts specifically on endothelial cells. It is possible that in other cell types, e.g., fibroblasts, epithelial cells, the deficiency of MAMs demonstrates reduced motility properties and angiogenesis. VEGFR2 is a potential endothelial cell marker, and data on VEGFR2 expression may also have therapeutic significance in view of the recent availability of VEGFR2 inhibitors. We screened VEGFR2 under FUNDC1-related MAMs regulation and discovered that FUNDC1 upregulated VEGFR2 expression in a Ca^2+^/SRF-dependent transcriptional manner. A critical question for future studies will be to determine whether FUNDC1-related MAMs formation in endothelial cell exclusively generate angiogenesis to accelerate tissue regeneration or repair under conditions. This will yield major breakthroughs in understanding the basic properties of an endothelial cell. Hopefully, MAMs will be new targets for chemotherapeutic interventions in angiogenesis.

FUNDC1 can interact with LC3 and act as a mitophagy receptor^20^. We have approved that manipulation of FUDNC1 could mimic MAMs expression, but the role of FUNDC1-related mitophagy in angiogenesis was still wide open. From our data, we surprisingly found that FUNDC1 knockdown had no effects on mitophagy in endothelial cells under non-stimulation conditions. We used mt-Keima, a ratiometric pH-sensitive fluorescent protein that is targeted into the mitochondrial matrix and monitor the mitophagy. Results showed that FUNDC1 knockdown did not alter the incidence of red mt-Keima puncta. To confirm this finding, we determined the co-localization of mitochondria and lysosomes. Consistently, FUNDC1 knockdown couldn’t alter the co-localization of the two organelles (Supplementary Fig. 8a, b). Removal of damaged mitochondria through autophagy requires mitophagy receptor proteins, induction of general autophagy, and activation of priming damaged mitochondria for selective autophagic recognition^40^. In addition to FUNDC1, mitochondrial priming is mediated either by the Pink1-Parkin signaling pathway or the mitophagic receptors Nix and Bnip3^40^. We think that the PTEN-induced putative kinase 1 (PINK1)-Parkin signaling pathway or Nix and Bnip3 mitophagy receptors under FUNDC1 deficiency condition may have a compensation effect on the damaged mitochondria clearance. Thus, deficiency of FUNDC1 is not sufficient to reduce mitophagy under angiogenic conditions.

MAMs have emerged as one important molecular platform for NLRP3 inflammasome activation. A recent study found hepatocyte-specific FUNDC1 ablation accumulated dysfunctional mitochondria and triggers inflammasome activation, which suppresses human hepatocellular carcinomas initiation^41^. Upon pro-inflammatory stimuli, NLRP3 recruits ASC (apoptosis-associated speck-like protein containing a CARD) and redistributes to the MAM fraction. NLRP3 oligomerizes and exposes its effector domain to interact with ASC, which in turn recruits pro-caspase-1. Finally, activated caspase-1 cleaves pro-IL-1β to generate mature IL-1β^42^. Evidences show that the assembly of the inflammasomes results in rapid activation of caspase-1, which disrupted the ability of endothelial precursor cells to differentiate into mature endothelial cells, thus retards angiogenesis^43^. Recently, mouse models have been described that dysregulated activation of the NLRP3 inflammasome led micro- and macroglia cells to a continuous activated state causing vascular leakage and retinal neovascularization in the diabetic retinopathy^44^. However, in endothelial cells, the precise role of MAMs-associated inflammasome activation in angiogenesis is still unknown. Although we postulated that intracellular Ca^2+^ homeostasis mediated by FUNDC1-dependent MAMs could influence the pathophysiological progress of angiogenesis, the effect of inflammasome status in MAMs is of interest to investigate in the future.

In summary, we have identified a new role for FUNDC1-related MAMs in mediating angiogenesis, through interfering with intracellular Ca^2+^ communication and regulating VEGFR2 expression. This mechanism may be crucial for the pathogenesis of physiological angiogenesis, and FUNDC1-dependent MAMs could represent a new therapeutic target for angiogenesis-related diseases.

## Methods

### Antibodies and reagents

VEGF-A165 was obtained from Sigma-Aldrich (St. Louis, MO; V5765). SiRNA for FUNDC1, IP3R1, VEGFR2, serum response factor (SRF), and controls were obtained from Santa Cruz Biotechnology (sc-36563, sc-91118, sc-42475, and sc-29318; Dallas, Texas). Antibodies against the following proteins were used as the primary antibodies: CD31 (BD Pharmingen, San Jose, CA; 550274; Cell Signaling Technology, Danvers, MA;77699); β-actin and GAPDH (Santa Cruz Biotechnology, Dallas, Texas; sc-47778 and sc-137179); PCNA, Calreticulin, Cyt C, VDAC1, mitofusin-2 (MFN2), SRF, phosphorylated (p) SRF, VEGFR1, VEGFR2, pVEGFR2, and VEGFR3 (Cell Signaling Technology, Danvers, MA; 13110, 12238, 4280,4661, 9482, 5147,4261, 2893, 2478, 2479, 3408); IP3R1 (Abcam, Cambridge, MA; ab5804; ThermoFisher, Waltham, MA, PA1-901); calnexin (Abcam, Cambridge, MA; ab22595); FUNDC1 (Aviva Systems Biology, San Diego, CA; ARP53281), FUNDC1 (Novus Biologicals, LLC, Littleton CO, NBP1-81063; EMD Millipore Corporation, Temecula, CA, ABC506).

### Endoplasmic reticulum and mitochondria contact analysis

Cells were labeled with MitoTracker® Green FM (ThermoFisher Scientific, Waltham, MA; 250 nm, Ex/Em490/516 nm, 7514) together with ER staining by endogenous calnexin antibody before confocal microscopic imaging with an LSM800 microscope (Carl Zeiss Microscopy Ltd, Cambridge, MA). Cell Images analyzing ER and mitochondrial co-localization were acquired using 3-dimensional deconvoluted stacks using the *Z*-stack application. Pearson correlation analysis was used to quantify the degree of co-localization between fluorophores ER and Mitochondria^45^. The Pearson’s correlation coefficient was analyzed using the built-in Carl Zeiss co-localization analysis module from the ZEN software, using the thresholding obtained from single-label control samples.

### Transmission electron microscopy

Endothelial cells were post-fixed for 2 h in a mixture of 0.8% potassium ferrocyanide and 2% osmium tetroxide in 0.1 M sodium cacodylate buffer. After dehydration in a graded series of acetone, the samples were embedded in Embed 812 resin, and ultrathin sections were cut and post-stained with uranyl acetate and lead citrate. The samples were observed and randomly imaged using a JEOL 1200EX transmission electron microscope (TEM; Tokyo, Japan). ER–mitochondrial contacts were quantified as described previously^21,24,34^. 100 pictures of each experimental group were obtained at ×7800 magnification. The images were analyzed using ImageJ (National Institutes of Health). The mitochondrial and ER membranes were delineated using the freehand tool. The selected areas were converted to masks and the perimeter of ER and mitochondria were calculated. Two independent investigators quantified the images blindly. For the MAM quantification (distance between ER/SR and mitochondria within 30 nm), we normalized the total ER connected to mitochondria to total ER perimeter or total mitochondrial perimeter.

### Endothelial cell capillary-like tube formation assay

Growth factor-reduced matrigel (356237, BD Biosciences, San Jose, CA) was pipetted into prechilled 24-well plates and polymerized at 37 °C. Endothelial cells were then collected and placed onto the matrigel layer. After 6 h, the endothelial cells were photographed using an inverted microscope (IX73, Olympus, Shinjuku-ku, Tokyo). Three independent experiments were performed. The tube network was quantified using ImageJ (National Institutes of Health, Bethesda, Maryland). Ten randomly selected fields were photographed in each group. Tube length was calculated by drawing a line along each tube and measuring the length of the line in pixels using the ImageJ software. The average of five fields was taken as the value for each treatment.

### Endothelial spheroid capillary sprouting

The endothelial capillary sprouting assay was performed following established protocols^46^. To form spheroids, endothelial cells were cultured overnight in hanging drops in Endothelial Basal Medium-2 (EBM-2) with methylcellulose (Sigma-Aldrich; 20% volume of a 1.2% solution of methylcellulose viscosity). Spheroids were then fixed with 4% paraformaldehyde and imaged under phase-contrast illumination with an Olympus microscope (Shinjuku-ku, Tokyo). Phase-contrast images were used to quantify the total sprout length (cumulative length of all sprouts on a spheroid).

### Generation of the endothelial cell-specific FUNDC1 knockout mice

The FUNDC1 gene was located in Chromosome X. The Cre*Cdh5*^*+/*^^−^ mice were purchased from Jackson Laboratory (Bar Harbor, ME). Littermate male *FUNDC1*^*f/Y*^*Cdh5*^−^ mice were considered as control mice. All mice were C57BL/6J background and housed in temperature-controlled cages with a 12-h light-dark cycle and given free access to water and food. All experimental procedures were approved by the Institutional Animal Care and Use Committee of Union Hospital, Tongji Medical College, Huazhong University of Science and Technology, and were performed in accordance with relevant institutional and national guidelines and regulations.

### In vivo matrigel plug assay

The formation of new vessels in vivo was evaluated using a matrigel plug assay. Briefly, Ice-cold matrigel (0.5 mL/plug; BD Biosciences) was mixed with 250 ng/mL FGF2, 0.0025 Unit/mL heparin, and 100 ng/mL VEGF was injected subcutaneously into the abdomen of 6-week-old *FUNDC1*^*f/Y*^*Cdh5*^−^ mice and *FUNDC1*^*f/Y*^*Cdh5*^*+*^ mice. After 10 days, the skin of each mouse was removed to expose an intact matrigel plug. CD31 staining was then performed to identify the formation and infiltration of new functional microvessels. Hemoglobin (Hb) content in the matrigel plugs was measured using the Drabkin reagent (Sigma-Aldrich, St. Louis, MO) for quantification of blood vessel formation.

### Retina dissection, processing, and staining

Mice were killed and their eyes were enucleated in phosphate-buffered saline (PBS). The eyes were then fixed in 4% paraformaldehyde for 1 h at 4 °C, after which the retinas were removed, washed with PBS, and permeabilized with PBS containing 1% Triton X-100 for 1 h. The retinas were subsequently incubated overnight in 0.1% Tween and 10% normal goat serum (BioGenex, Fremont, CA) in PBS at 4 °C, followed by incubation for 1 h in Alexa Fluor 597-conjugated isolectin GS-IB4 solution (1:100) at 4 °C. Flat-mounted retinas were analyzed using an LSM800 confocal fluorescence microscope (Carl Zeiss Microscopy, Cambridge, MA). Quantification was performed by researchers unaware of the mouse’s genotype.

### Tumor inoculation model

6- to 8-week-old male *FUNDC1*^*f/Y*^*Cdh5*^*+*^ mice and their control littermates underwent allograft inoculation. Lewis lung carcinoma (LLC) cells (1 × 10^6^ cells) were suspended in 200 μL Hank’s balanced salt solution and injected subcutaneously in the flank of each animal. At the endpoint, host mice were euthanized and their tumors were removed. The tumors were weighed and then fixed in 4% paraformaldehyde, embedded in Tissue-Tek O.C.T. Compound (VWR, Radnor, PA), frozen on dry ice, sectioned, and mounted on glass slides. Staining with anti-CD31 antibody (565629, BD Biosciences, San Jose, CA) was used for qualitative identification of endothelial cells in histological tissue sections.

### Murine model of hind limb ischemia

Male *FUNDC1*^*f/Y*^*Cdh5*^*−*^ and *FUNDC1*^*f/Y*^*Cdh5*^+^ mice at 8–12 weeks of age were used for these experiments. Mice were anesthetized with an intraperitoneal injection of ketamine (150 mg/kg) and xylazine (10 mg/kg), and fixed on the back. A 1 cm operative incision, vertical to the inguinal ligament in the right hind limb, was exposed, and the external iliac artery was clearly isolated and ligated with the 8/0 polypropylene suture (Premilene, Melsungen AG). Blood perfusion in the footpad was assessed by a laser Doppler system (Perimed AB, Sweden) preoperatively, postoperatively and on day 10.

### Immunoprecipitation and western blotting

After treatment, cells were lysed in RIPA lysis buffer (sc-24948, Santa Cruz Biotechnology, Dallas, Texas). For immunoprecipitation, 300 µg of protein extracts were incubated with the indicated antibodies against Flag (Cell signaling technology, 14793), HA (Cell signaling technology, 3724), or unspecific IgG at 4 °C overnight, and protein-A/G agarose was added for another 2–3 h at 4 °C. The immunoprecipitates were pelleted by centrifugation at 12,000 × *g* for 30 s and washed 3 times with RIPA lysis buffer. The pellets were suspended in SDS gel loading buffer and subjected to western blot assays. Western blot analysis was performed as described previously^47–49^. Briefly, the tissues and cells were homogenized, separated by SDS-PAGE, and then transferred to nitrocellulose membranes. Blots were probed overnight with primary antibodies at 4 °C. The membranes were extensively washed and then incubated with the appropriate secondary antibody at room temperature for 2 h. Signals were captured with a Kodak X-Omat 2000A film developer (Rochester, NY).

### Total RNA isolation and real-time PCR analysis

Total RNA was extracted from cells with QIAzol and purified using the QIAGEN RNeasy Mini Kit (74104; QIAGEN, Germany). Total RNA (2 μg) was reverse-transcribed with iScript cDNA Synthesis Kit (Bio-Rad, Hercules, CA). Real-time PCR was performed using Brilliant II SYBR Green qPCR Master Mix (Stratagene, San Diego, CA) and the Bio-Rad Real-Time PCR System. Results were normalized to *18s rRNA*. Sequences of primers are available in Supplementary Table 1.

### Chromatin immunoprecipitation

Chromatin immunoprecipitation (ChIP) assay was performed using the SimpleChIP® Enzymatic Chromatin IP Kit (Cell Signaling Technology, Danvers, MA). Briefly, cells were crosslinked with 1% formaldehyde and neutralized with 0.125 M glycine. Cell lysates were digested by micrococcal nuclease, then sonicated. The proteins were immunoprecipitated with antibody to SRF or rabbit IgG (as a control). After complete washing, immunoprecipitated DNA was eluted with elution buffer and reverse crosslinked overnight at 65 °C. The DNA was purified and quantified by real-time PCR. Enrichment was calculated relative to input.

### Luciferase activity assay

The pGL3 Luciferase Reporter Vectors (Promega, Madison, WI) were used for constructing luciferase vectors. To identify the SRF-binding site in the human VEGFR2 promoter, a series of 5′ VEGFR2 promoter deletions (pGL-VEGFR2-40, pGL-VEGFR2-125, pGL-VEGFR2-280, pGL-VEGFR2-420, and pGL-VEGFR2-570) was constructed by amplifying the VEGFR2 promoter region with different forward primers. The putative binding site of SRF, which is located at −50 bp from the human VEGFR2 promoter, was deleted by site-directed mutagenesis using a QuikChange II Kit (Stratagene, San Diego, CA), according to the manufacturer’s protocol. Human umbilical vein endothelial cells (HUVECs) were transfected with the plasmids, according to the manufacturer’s recommendation. Luciferase activity was measured using the Dual-Luciferase Reporter Assay System (Promega, Madison, WI).

### Mitochondrial-associated membranes fractionation isolation

The subcellular fractionation was performed based on published protocols^50^. HUVECs were harvested and washed with PBS by centrifugation at 600 × *g* for 5 min. The cell pellet was resuspended in starting buffer (225 mM mannitol, 75 mM sucrose, and 30 mM Tris-HCl pH 7.4) and homogenized. The homogenate was centrifuged three times at 600 × *g* for 5 min to remove nuclei and unbroken cells. The crude mitochondria were pelleted by centrifugation at 10,000 × *g* for 10 min. To separate MAM and pure mitochondria fractions, the pellet was resuspended in MRB buffer (250 mM mannitol, 5 mM HEPES and 0.5 mM EGTA, pH 7.4) and layered on top of different concentrations of Percoll gradient (225 mM mannitol, 25 mM HEPES, 1 mM EGTA pH 7.4 and 30% or 15% Percoll). After centrifugation at 95,000 × *g* for 30 min, two dense bands containing either the pure mitochondria or MAM fraction were recovered and washed twice with MRB buffer by centrifugation at 6300 × *g* for 10 min to remove residual Percoll and residual contamination. MAM was pelleted by centrifugation at 100,000 × *g* for 1 h and resuspended in Lysis Buffer (50 mM Tris, 150 mM NaCl, 1% Triton X-100, 10 mM EDTA, pH 7.2, and protease inhibitor cocktail). Protein concentrations were determined by Bradford assay. Equal amount of proteins was loaded on 4–20% gradient SDS-PAGE gels (Bio-Rad, Hercules, CA) and immunoblotting was carried out.

### Immunofluorescence staining and fluorescence confocal microscopy

Tissue or cell chamber slides were washed with PBS, fixed in 4% paraformaldehyde, permeabilized with 0.1% Triton X-100, and blocked with 1% bovine serum albumin. The slides were then incubated with primary antibodies, followed by incubation with Alexa Fluor-conjugated secondary antibodies. Fluoroshield mounting medium with DAPI (VECTOR Laboratories, Burlingame, CA) was used to cover the slides. Images were visualized under an Olympus inverted microscope equipped with a charge couple camera.

### Isolation of endothelial cells from a tumor or skeletal muscle

Endothelial cells were isolated from freshly resected ischemic skeletal muscle and Lewis lung carcinoma as described previously^51,52^. Briefly, tissues were was cut into pieces and digested with collagenase. CD31 microbeads (MACS Miltenyi Biotec, Cat No. 130-097-418, San Diego, CA) were used to separate ECs from the tissue homogenates. First, the CD31-positive cells were magnetically labeled with CD31 microbeads. Then, the cell suspension was loaded onto a MACS Column, which was placed in the magnetic field of a MACS separator. The magnetically labeled CD31-positive cells were retained within the column. After removing the column from the magnetic field, the magnetically retained CD31-positive cells were eluted as the positively selected cell fraction. Then CD31 microbead-labeled cells were cultured in DMEM and 10% FBS and used for expression analysis.

### Mitochondria–ER linker construction

Adenovirus-expressing linker and control were a gift from Dr. Ana Paula, at the Harvard School of Public Health. The synthetic ER–Mitochondria linker (Linker) plasmid was constructed in the previous reports^6^. Briefly, a construct that encodes the OMM targeting sequence of mAKAP1 at the N terminus, the monomeric red fluorescent protein (mRFP) in the middle, and the ER targeting sequence of yUBC6 at the C terminus (mAKAP1 [34–63]-mRFP-yUBC6, OMM–ER linker) was prepared. Based on the size of the fluorescent protein (4.2 × 2.4 nm), the maximal length of this construct is <5 nm. As a control, the above construct was also prepared without the ER targeting sequence (mAKAP1 [34–63]-mRFP). The linker used in these experiments was designed to link ER and mitochondria to a 20–30 nm proximity. For in vivo linker experiments, the construct was cloned into an adenovirus backbone (Vector Biolabs) under the CMV promoter. Adenovirus was amplified in 293 A cells, purified twice using CsCl column, desalted, and 1 × 10^10^ IFU/mouse were injected intravenously. For histological analysis, muscle or tumor tissues were fixed in 10% formalin solution and sectioned for immunofluorescence staining. For TEM analysis, CD31-positive endothelial cells were isolated after adenovirus infection (Supplementary Fig. 9).

### Recombinant adenovirus construction and infection

Adenovirus-expressing empty vector (Ad-*Null*), Ad-Flag-tagged human *FUNDC1* (Ad-Flag-*FUNDC1*), and Ad-Flag-tagged human *VEGFR2* were obtained from Abm (Applied Biological Materials, Canada). HUVECs were infected with adenovirus at a multiplicity of infection of 10, no cellular toxicity occurred. For in vivo experiment, the adenoviruses were injected intravenously in a titer of 1 × 10^10^ IFU/mouse.

### siRNA transfection

HUVECs were transfected with FUNDC1, IP3R1, SRF, or VEGFR2 siRNA in OPTI-MEM reduced-serum media (GIBCO, 31985) using Lipofectamine™ RNAiMax transfection reagent (Life Technologies, 13778150).

### Peptide synthesis and delivery

The inhibitory peptides for blocking the interaction between FUNDC1 and IP3R1 were designed based on the degree of homology in the conserved interactive region among different IP3R isoforms. The 11 amino acid long peptide (YGRKKRRQRRR) from Tat protein transduction domain served as a cell-penetrating peptide. Thus, inhibitory peptides were chemically synthesized by linking with biotin-labeled cell-penetrating peptide at the N terminus and conjugating with FITC at C terminus. The purity of synthesized peptides was >95%, verified by mass spectrometry and HPLC. Synthesized peptides were coupled to NHS/PEG/DSPE [*N*-hydroxysuccinimidocarboxyl/polyethylene glycol (PEG; average molecular weight, 3000)-derived/distearoylphosp hatidylethanolamine] in a 1:1.5 molar ratio. Peptidyl-PEG/DSPE was transferred to preformed liposomes after coincubation at a temperature above the transition temperature of the lipid bilayer. The liposomes had a particle size ranging from 65 to 75 nm in diameter. There were 300–500 peptide molecules perliposome, computed as described previously^53^. For in vitro experiment, HUVECs were mixed with the peptide concentration at 20 μM. For in vivo experiment, the mice received an intravenous injection of the indicated peptides (10 mg/kg/3 days) as showed in Supplementary Fig. 10.

### Intracellular Ca^2+^ measurement

Endothelial intracellular Ca^2+^ was measured in Fluo-4 AM loaded endothelial cells on a Carl Zeiss confocal microscope. An argon laser (488 nm) at 50% power was used for excitation, and the emission band was 510–530 nm. Quantitative analysis of endothelial [Ca^2+^]cyto at the individual endothelial cell level was conducted using manually selected regions of interests (ROIs) along the microchannels. Each ROI covered the area of one individual endothelial cell, as indicated by the fluorescence outline. The changes in endothelial [Ca^2+^]cyto were quantified by calculating the mean fluorescence intensity (FI) of each stack of ROIs after the subtraction of the background autofluorescence. The percent change in FI was expressed as FI/FI_0_, where FI_0_ was the initial baseline FI of fluo-4.

### Quantification of mitophagy

Lysosomal engulfment of mitochondria was calculated by counting the number of co-localized lysosomes and mitochondria per cell detected by confocal co-localization of LysoTracker Red and MitoTracker Green^54^. Mt-Keima is a ratiometric pH-sensitive fluorescent protein that is targeted into the mitochondrial matrix. Mitochondria-targeted mKeima-Red expression plasmid (pMT-mKeima-Red, #AM-V-251, MBL Medical & Biological Laboratories Co. ltd. Woburn, MA) was transfected to HUVECs. After 48-hour transfection, cells were subjected to confocal microscopy using dual-excitation at 488 (pH 7) and 562 (pH 4) nm lasers, with 620/29 nm emission filters.

### Statistics

Statistical analysis was performed using SPSS Statistics (IBM SPSS Statistics for Windows, Version 22.0. Armonk, NY, USA) and Prism 6.0 software (GraphPad Software, San Diego, CA, USA). Data are expressed as mean ± standard deviation (SD). A two-tailed unpaired Student’s *t*-test was used for two-group comparisons. For comparisons of more than two groups, one-way analysis of variance (ANOVA) was used for normally distributed data, and the Kruskal–Wallis test was used for non-normal or small samples. A value of *p* < 0.05 was considered statistically significant.

### Reporting summary

Further information on research design is available in the Nature Research Reporting Summary linked to this article.

### Supplementary information

Supplementary information

Reporting Summary

### Source data

Source data

## Data Availability

The data that support the findings of this study are available within the article and its supplementary information or from the corresponding authors on reasonable request. Source data are provided with this paper.
