# Author Correction: FUNDC1-dependent mitochondria-associated endoplasmic reticulum membranes are involved in angiogenesis and neoangiogenesis

**DOI:** 10.1038/s41467-024-48817-w

**Published:** 2024-05-29

**Authors:** Cheng Wang, Xiaoyan Dai, Shengnan Wu, Wenjing Xu, Ping Song, Kai Huang, Ming-Hui Zou

**Affiliations:** 1 Center for Molecular and Translational Medicine, Georgia State University, Atlanta, Georgia USA; 2 Clinic Center of Human Gene Research, Union Hospital, Tongji Medical College, Huazhong University of Science and Technology, Wuhan, China

**Keywords:** Endoplasmic reticulum, Mitochondria, Angiogenesis, Tumour angiogenesis

Correction to: *Nature Communications* 10.1038/s41467-021-22771-3, published online 10 May 2021

The original version of this Article omitted from the author list Dr Ming-Hui Zou, from the Center for Molecular and Translational Medicine of Georgia State University, who is now listed as a corresponding author. Additionally, the following has been added to the Author contribution list: M.H. Zou conceived/supervised the project, provided the funding, and edited/revised the manuscript. The following has been added to the Acknowledgements section: This study was supported in part by the grants from the National Heart, Lung, and Blood Institute (NHLBI) (HL079584, HL080499, HL089920, HL110488, HL128014, HL132500, HL137371, HL142287, and HL140954 to Dr. Zou); National Cancer Institute (NCI) (CA213022 to Dr. Zou); and National Institute on Aging (NIA) (AG047776 to Dr. Zou). Dr. Zou is the Eminent Scholar in Molecular and Translational Medicine of the Georgia Research Alliance. Dr. Cheng Wang was supported by an American Heart Association postdoctoral fellowship award (19POST34450166). Dr. Kai Huang’s lab was supported by the National Natural Science Foundation of China (81900268), Major key technology research project of the Science and Technology Department in Hubei Province (2016ACA151), Key projects of Huazhong University of Science and Technology (2016JCTD107), and Wuhan Science and Technology Plan Project (2018060401011328).

This has been corrected in both the HTML and PDF versions of the Article.

