# Supplementary material for: FUNDC1-dependent mitochondria-associated endoplasmic reticulum membranes are involved in angiogenesis and neoangiogenesis
Source: Nat Commun. 2021 May 10;12:2616. doi: 10.1038/s41467-021-22771-3 (PMC8110587; doi:10.1038/s41467-021-22771-3)
Supplement: Supplementary file 1 — Supplementary information [file 41467_2021_22771_MOESM1_ESM.pdf]

SUPPLEMENTAL MATERIAL

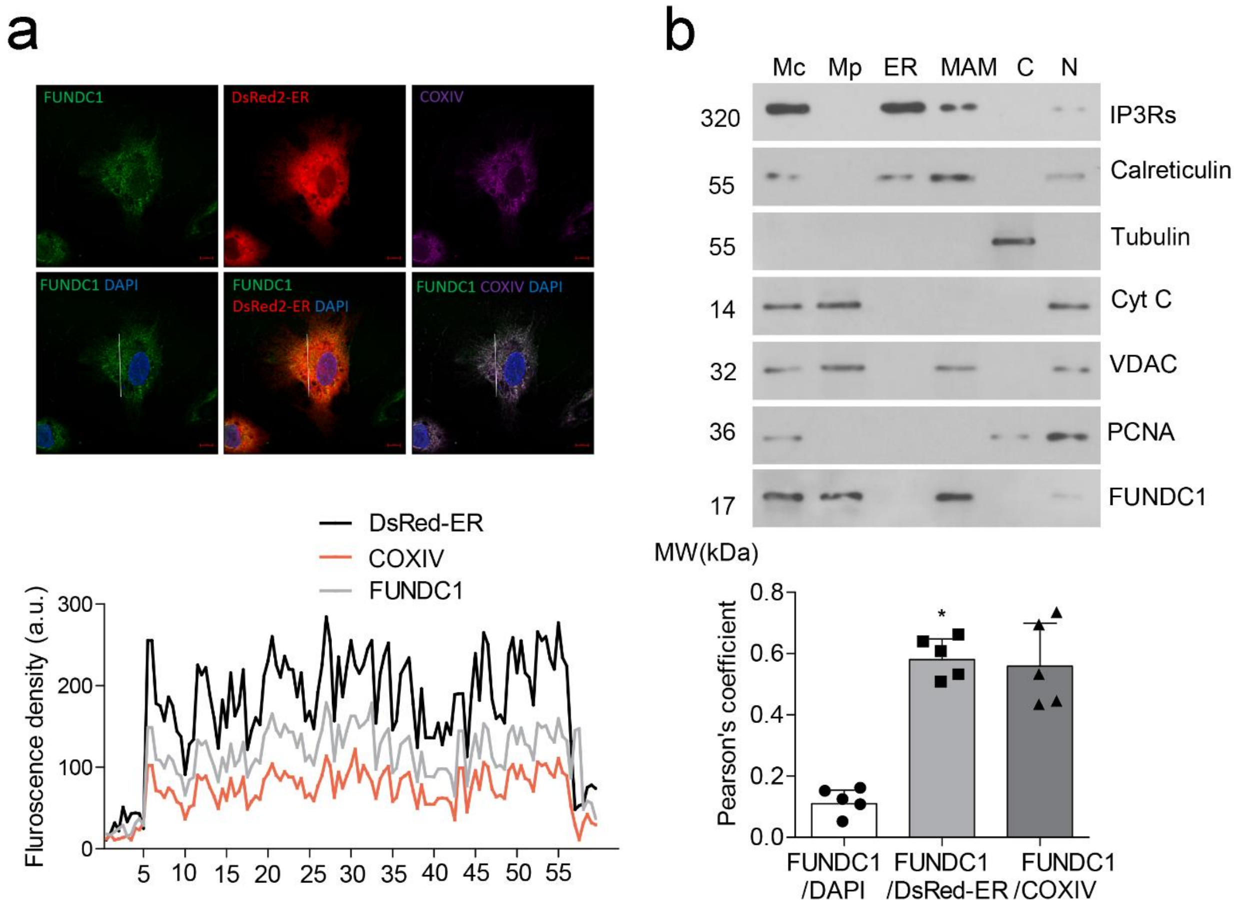

Supplementary Figure 1. (**a**) Co-localization of FUNDC1 with mitochondria and ER. Mitochondria was labeled with COXIV; ER was labeled with pDsRed2-ER; FUNDC1 was indicated by anti-FUNDC1; Nucleus was stained by DAPI. Representative confocal microscopy images were shown. Traces of fluorescence intensity spatial profiles through the white line for MitoTracker, DsRed2-ER, and FUNDC1 fluorescence. Quantitation of FUNDC1/DAPI, FUNDC1/ER and FUNDC1/mitochondria colocalization using the Pearson's coefficient. (*n*=5 independent experiments) Scale bar 10μm. (**b**) Western blot analysis of the protein levels of FUNDC1 in crude mitochondria (Mc), as well as, pure mitochondria (Mp), MAMs, ER, cytosolic (C) and nuclear fraction (N) from HUVECs. Calreticulin was used as an ER marker, cytochrome c (Cyt C) and voltage dependent anion channel 1 (VDAC1) was used as a mitochondrial marker, Tubulin was used as cytosol marker, PCNA were used as nuclear marker and inositol 1,4,5-

trisphosphate type 1 receptor (IP3R1) was used as a MAMs component (*n*=5 independent experiments), Statistical significance was assessed using one-way ANOVA with post hoc multiple comparisons test for comparing multiple groups. **p* < 0.05. All values are mean ± S.D.

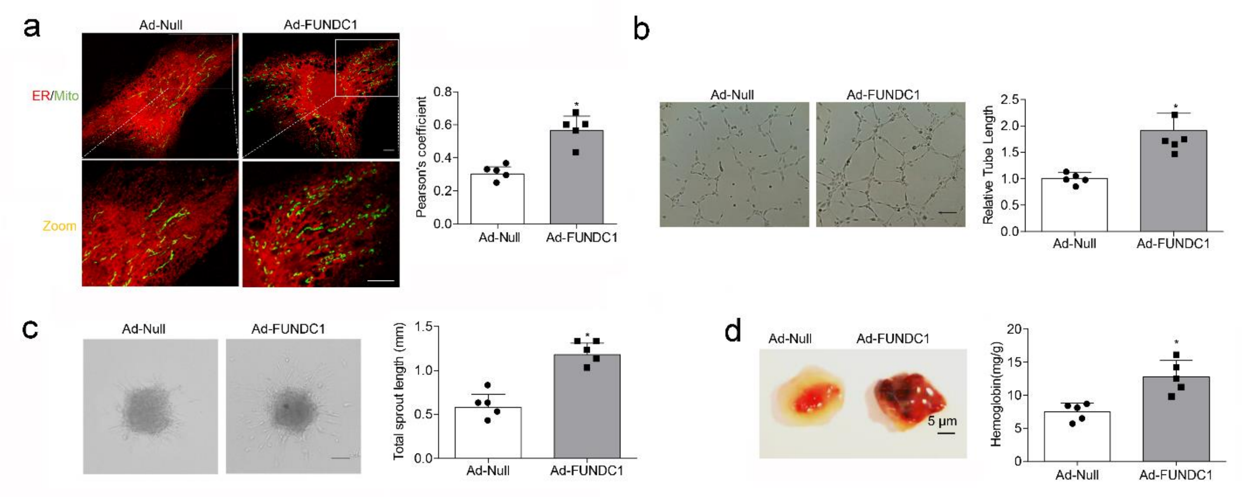

Supplementary Figure 2. (**a**) HUVECs infected with Ad-Null or Ad-FUNDC1 for 24 hours. Representative Confocal images for ER and mitochondria are shown. Quantitation of ER-mitochondria contacts using the Pearson's coefficient. (*n*=5 independent experiments) Scale bar 10μm. (**b**) HUVECs pre-infected with adenovirus coding Null or FUNDC1 were plated onto matrigel for 6 hours. Representative images of tube formation are shown. Tube number/field in 10 random microscopic fields per group were measured by NIH ImageJ (*n*=5 independent experiments). Scale bar 100μm. (**c**) Three-dimensional spheroids and representative images of spheroids sprouting was analyzed (*n*=5 independent experiments). Scale bar 100μm. (**d**) Matrigel containing VEGF was injected subcutaneously into WT mice that had received intravenous administration of FUNDC1 adenoviruses 24 h before. After 10 days, matrigel plugs were removed for analysis of new vessel formation by histological and hemoglobin assay (*n*=5 mice/group). Quantification of Hb (mg/ml) extracted from matrigel plugs from the mice. Statistical significance was assessed using two-tailed t-tests for two groups and presented as follows: **p* < 0.05. All values are means ± S.D.

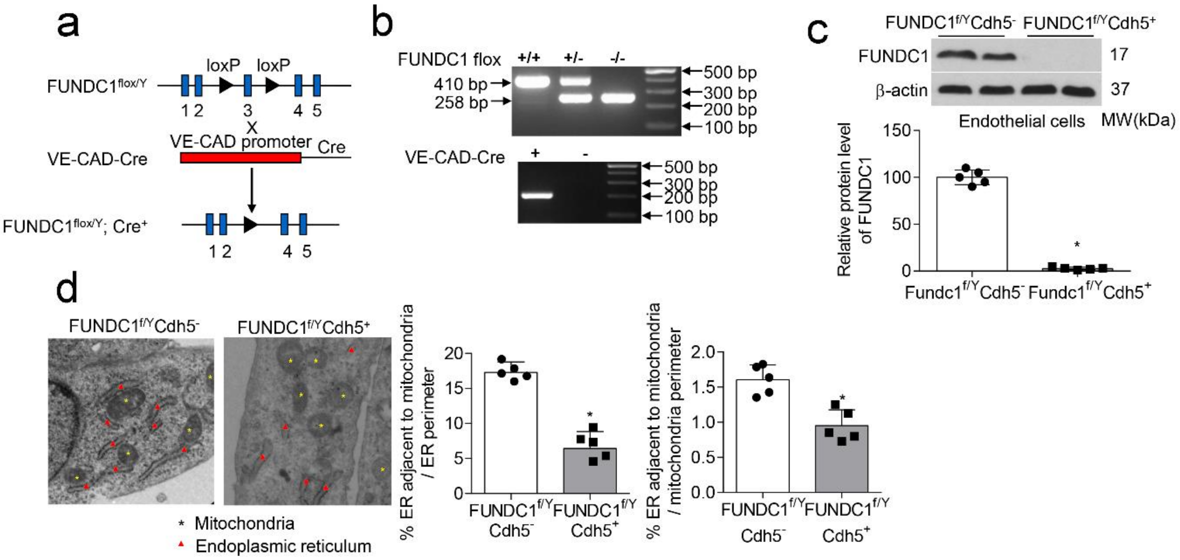

Supplementary Figure 3. Generation of endothelial-specific FUNDC1 knockout (KO) mice. (**a**) Endothelial FUNDC1 KO system. (**b**) Genotyping of *FUNDC1*$^{f/Y}$*Cdh5*$^{-}$ and *FUNDC1*$^{f\,/Y}$*Cdh5*$^{+}$ mice (*n*=5 independent experiments). (**c**) Western blot analysis of FUNDC1 levels in endothelial cells (*n*=5 mice/group). (**d**) Representative transmission electron microscope (TEM) images of the ER and mitochondrial morphology from endothelial cells of *FUNDC1*$^{f/Y}$*Cdh5*$^{-}$ and *FUNDC1*$^{f/Y}$*Cdh5*$^{+}$ mice (*n*=5 mice/group). Statistical significance was assessed using two-tailed t-tests for two groups and presented as follows: *$p < 0.05$. All values are means ± S.D.

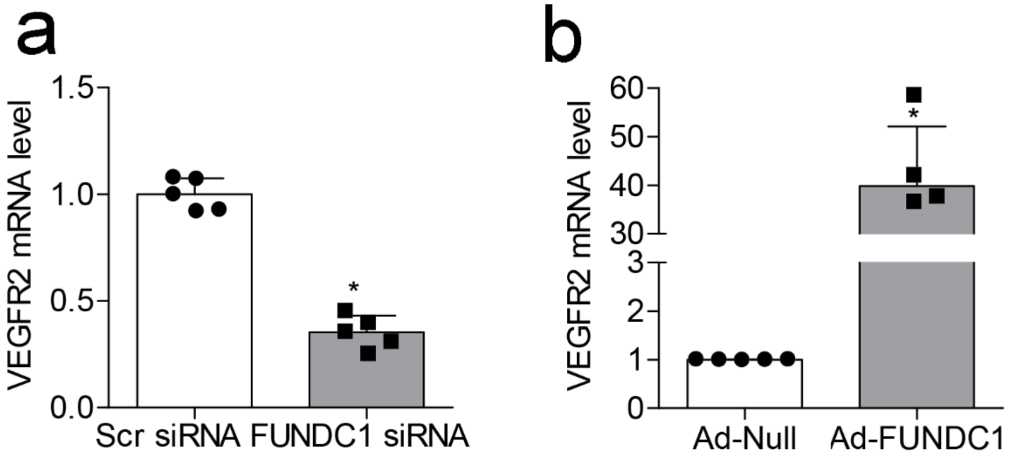

Supplementary Figure 4. (**a**) Human umbilical vein endothelial cells (HUVECs) were transfected with scrambled (Scr) siRNA or FUN14 domain containing 1 (FUNDC1) siRNA for 24 h, after which the mRNA levels of vascular endothelial growth factor receptor 2 (VEGFR2) were determined by RT-qPCR assay (*n*=5 independent experiments). (**b**) HUVECs were infected with Ad-Null or Ad-FUNDC1 for 24 h, after which the mRNA levels of VEGFR2 were determined by RT-qPCR assay (*n*=5 independent experiments). Statistical significance was assessed using two-tailed t-tests for two groups and presented as follows: *$p < 0.05$. All values are means ± S.D.

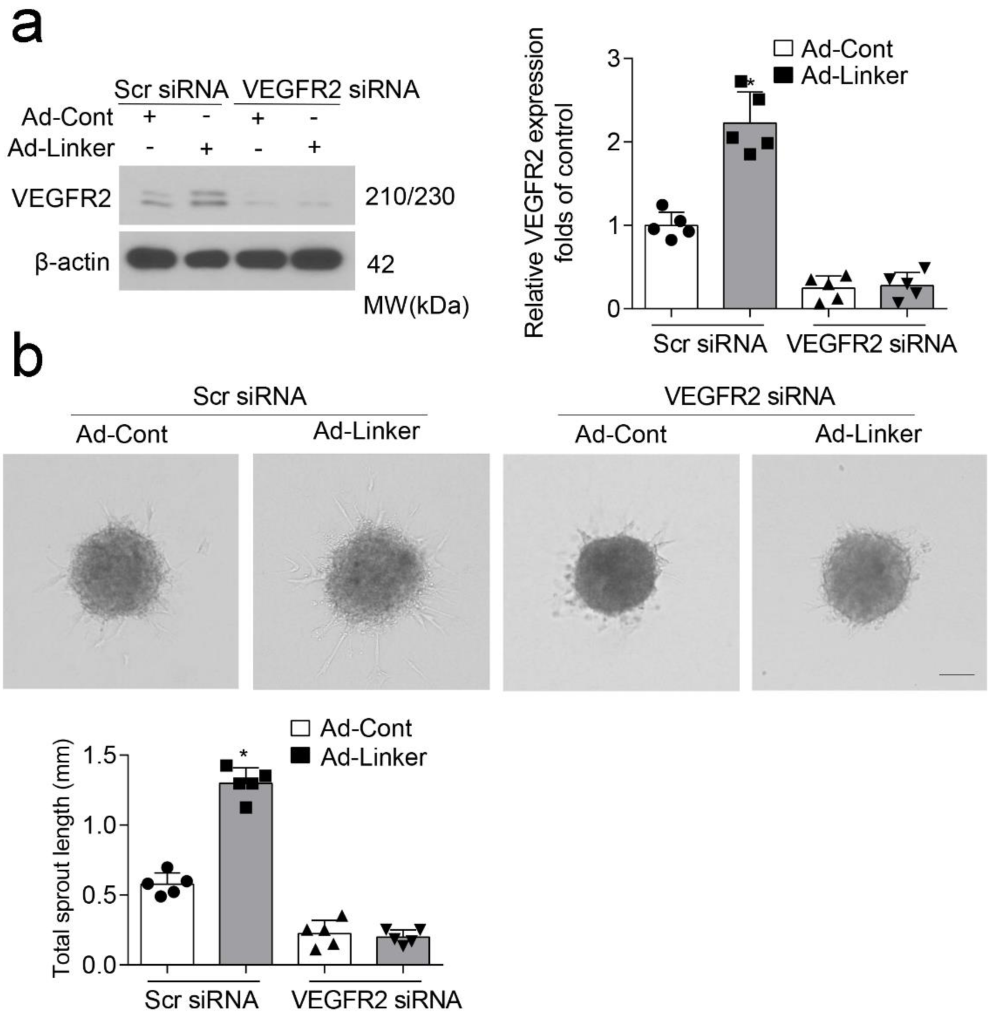

Supplementary Figure 5. HUVECs were transfected with Scr siRNA or VEGFR2 siRNA for 24 hours, and then infected with adenovirus encoding control (Cont) or Linker for another 24 hours. (**a**) The protein level of VEGFR2 was determined by western blot

assay (n=5 independent experiments). (**b**) Representative images of spheroid sprouting were shown. Sprouting length in 6 random microscopic fields per group were measured by NIH ImageJ and statistically analyzed. (n=5 independent experiments) Scale bar 100μm. Statistical significance was assessed using one-way ANOVA with post hoc multiple comparisons test for multiple comparison and presented as follows: $*p < 0.05$. All values are means ± S.D.

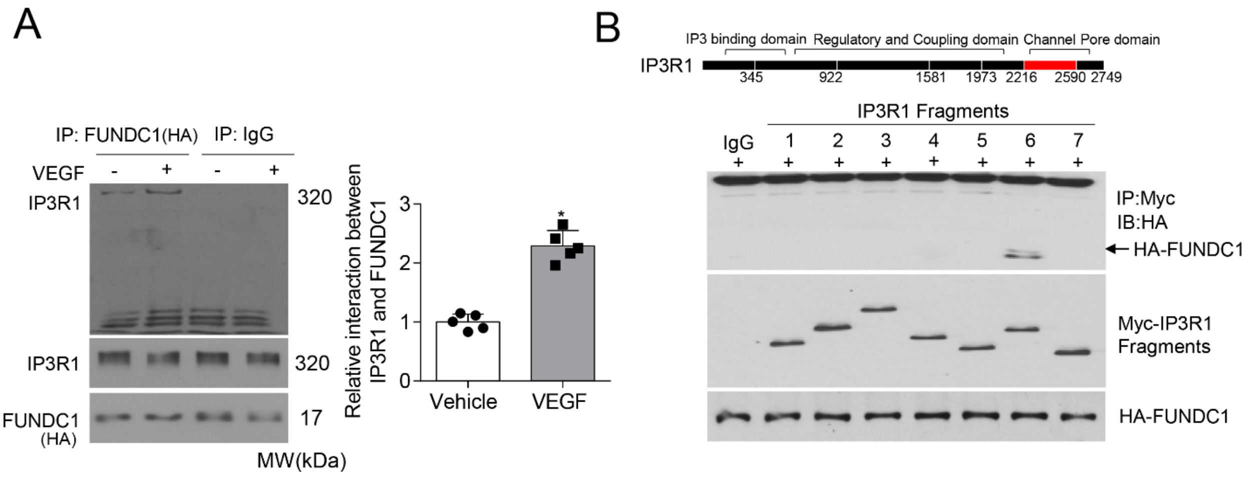

Supplementary Figure 6. (**a**) HUVECs were treated with vascular endothelial growth factor (VEGF; 30 ng/mL) for 6 hours, then subjected to immunoprecipitation with antibody against FUNDC1 to quantify the interaction of FUNDC1 and IP3R1 (n=5 independent experiments). (**b**) Diagram of Myc-tagged human IP3R1 with its domains. HEK293 cells were transfected with different Myc-tagged human IP3R1 fragments, along with HA-FUNDC1 for 36 hours (n=5 independent experiments). Whole cell extracts from HEK293 cells were subjected to immunoprecipitation assay with anti-Myc antibody followed by western blot assay using anti-HA antibody. Statistical significance was assessed using two-tailed t-tests for two groups and presented as follows: $*p < 0.05$. All values are means ± S.D.

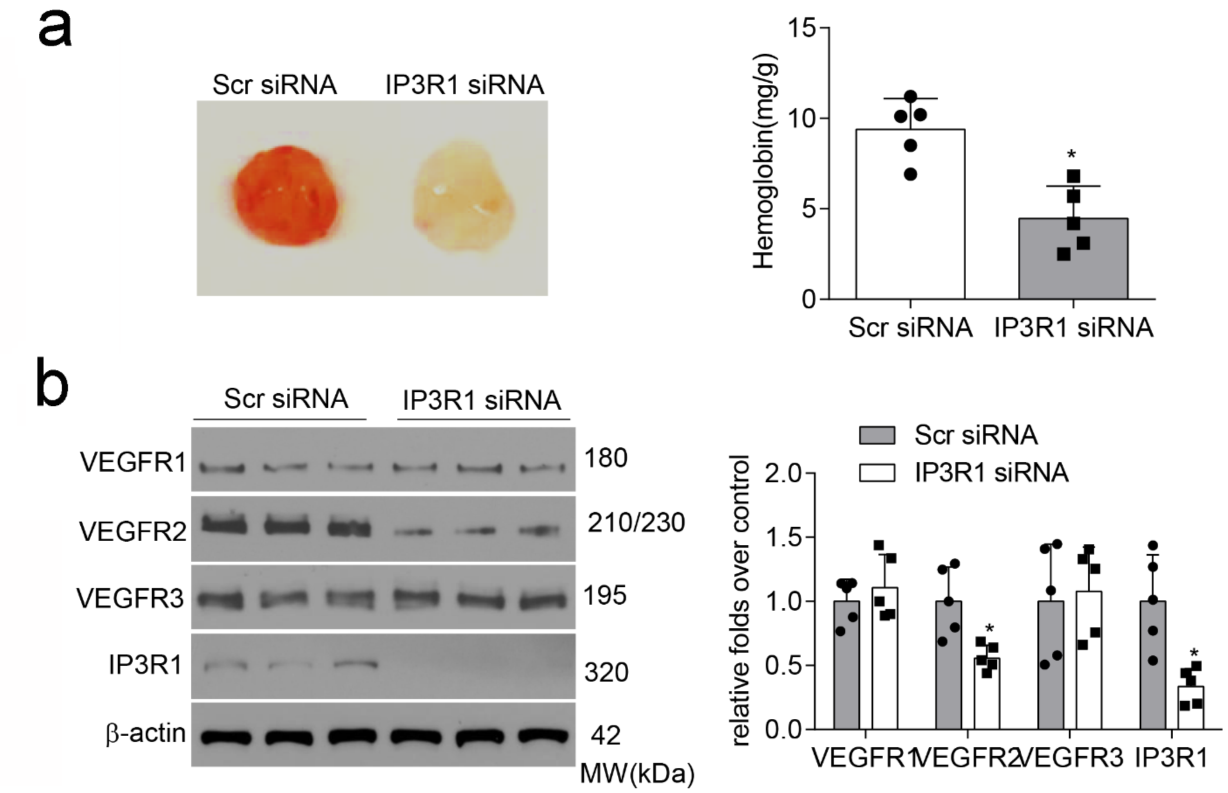

Supplementary Figure 7. (**a**) Matrigel containing VEGF was injected subcutaneously into 6-week-old mice that had received intravenous administration of Scr siRNA or IP3R1 siRNA containing liposomes (Life Technologies, Invivofectamine® 3.0, IVF3001; 7 mg/kg/every 5 days). After 10 days, matrigel plugs were removed for analysis of new vessel formation by histological and hemoglobin assay. Quantification of Hb (mg/ml) extracted from matrigel plugs from different groups (n=5 mice/group). (**b**) HUVECs were transfected with Scr siRNA or IP3R1 siRNA for 48 hours. Western blot analysis of VEGFR1, VEGFR2, VEGFR3 and IP3R1 expression (n=5 independent experiments). Statistical significance was assessed using two-tailed t-tests and presented as follows: $*p < 0.05$. All values are means ± S.D.

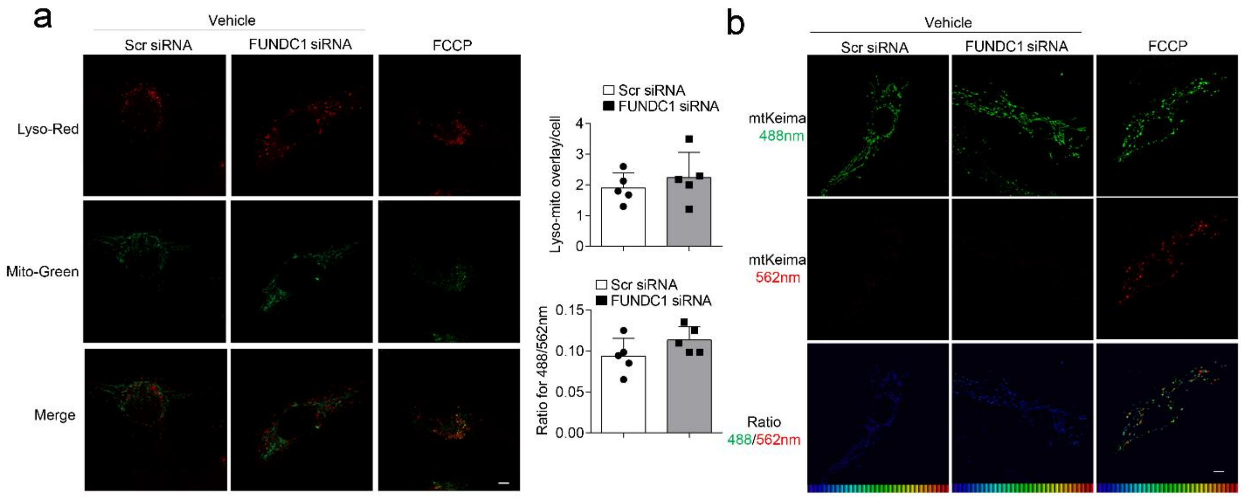

Supplementary Figure 8. HUVECs were transfected with Scr siRNA or FUNDC1 siRNA for 24 hours. (**a**) Mitochondria were labeled with MitoTracker Green, lysosomes were labeled with LysoTracker Red, and lysosomal-mitochondrial interactions were determined by confocal microscopy. Scale bars, 10 μm. Quantitative data of co-localization of lysosomes and mitochondria. Mitophagy in HUVECs induced by carbonylcyanide-4-(trifluoromethoxy)phenylhydrazone (FCCP) (20 μM, 2 h) was the positive control (*n*=5 independent experiments). (**b**) Mitophagy was indicated by mt-Keima using confocal microscopy. (*n*=5 independent experiments) Scale bars, 10 μm. Mitophagy in HUVECs was induced by carbonylcyanide-4-(trifluoromethoxy) phenylhydrazone (FCCP) (20 μM, 2 h) and served as a positive control. Statistical significance was assessed using two-tailed t-tests for two groups and one-way ANOVA with post hoc multiple comparisons test for multiple groups. *$p < 0.05$. All values are mean ± S.D.

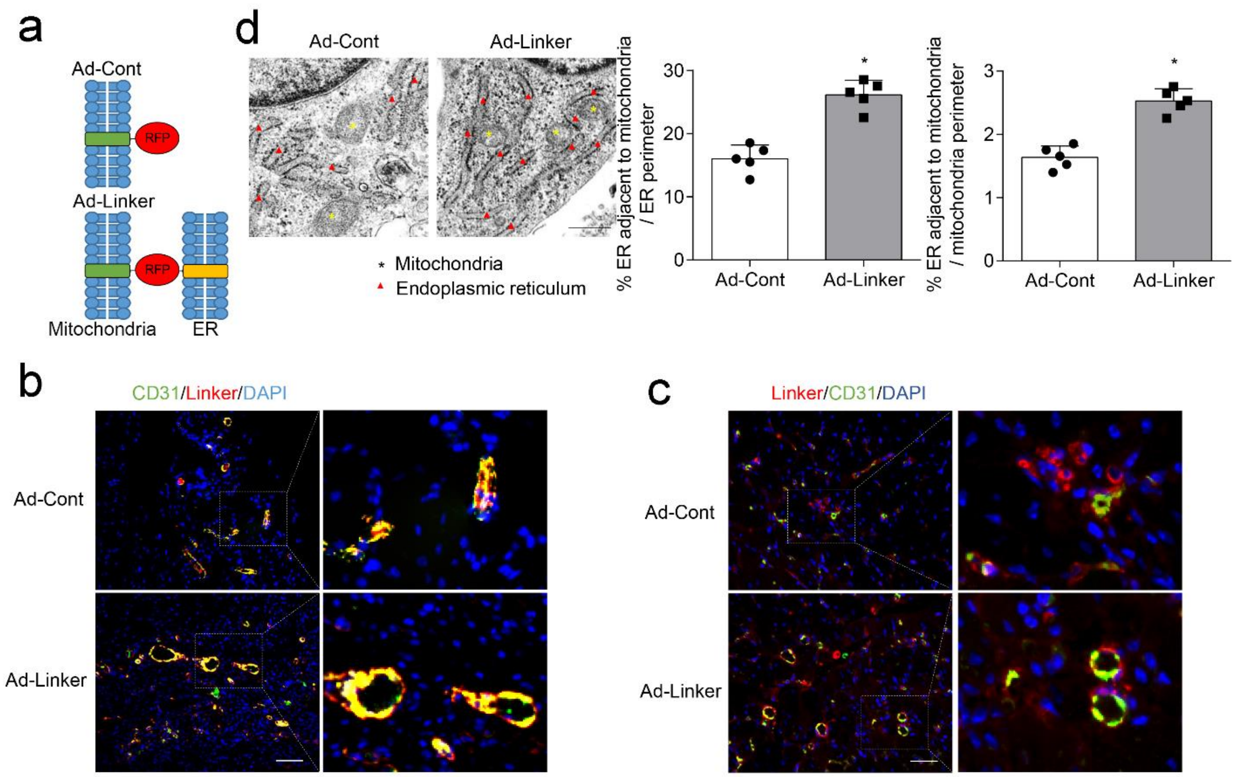

Supplementary Figure 9. (**a**) Schematic illustrating the mitochondrial-ER synthetic linker. The construct encodes a monomeric red fluorescent protein (RFP) fused to the OMM targeting sequence of mAKAP1 at the N terminus and the ER targeting sequence of yUBC6 at the C terminus. (**b**) CD31 and Control/Linker immunostaining of matrigel plugs implanted into $FUNDC1^{f/Y}Cdh5^{-}$ and $FUNDC1^{f/Y}Cdh5^{+}$ mice for 10 days. (*n*=5 mice/group) Scale bar, 100 µm. (**c**) Representative images showing CD31 and Control/Linker immunostaining in gastrocnemius muscle on day 10 after artery ligation. (*n*=5 mice/group) Scale bar, 100 µm. (**d**) Representative transmission electron microscopy images of ER and mitochondrial morphology from CD31-positive endothelial cells. Bar graph illustrating quantitation of ER length adjacent to mitochondria normalized by total ER length and by mitochondrial perimeter (*n*=5 mice/group). Statistical significance was assessed using two-tailed t-tests for two groups. *$p < 0.05$. All values are mean ± S.D.

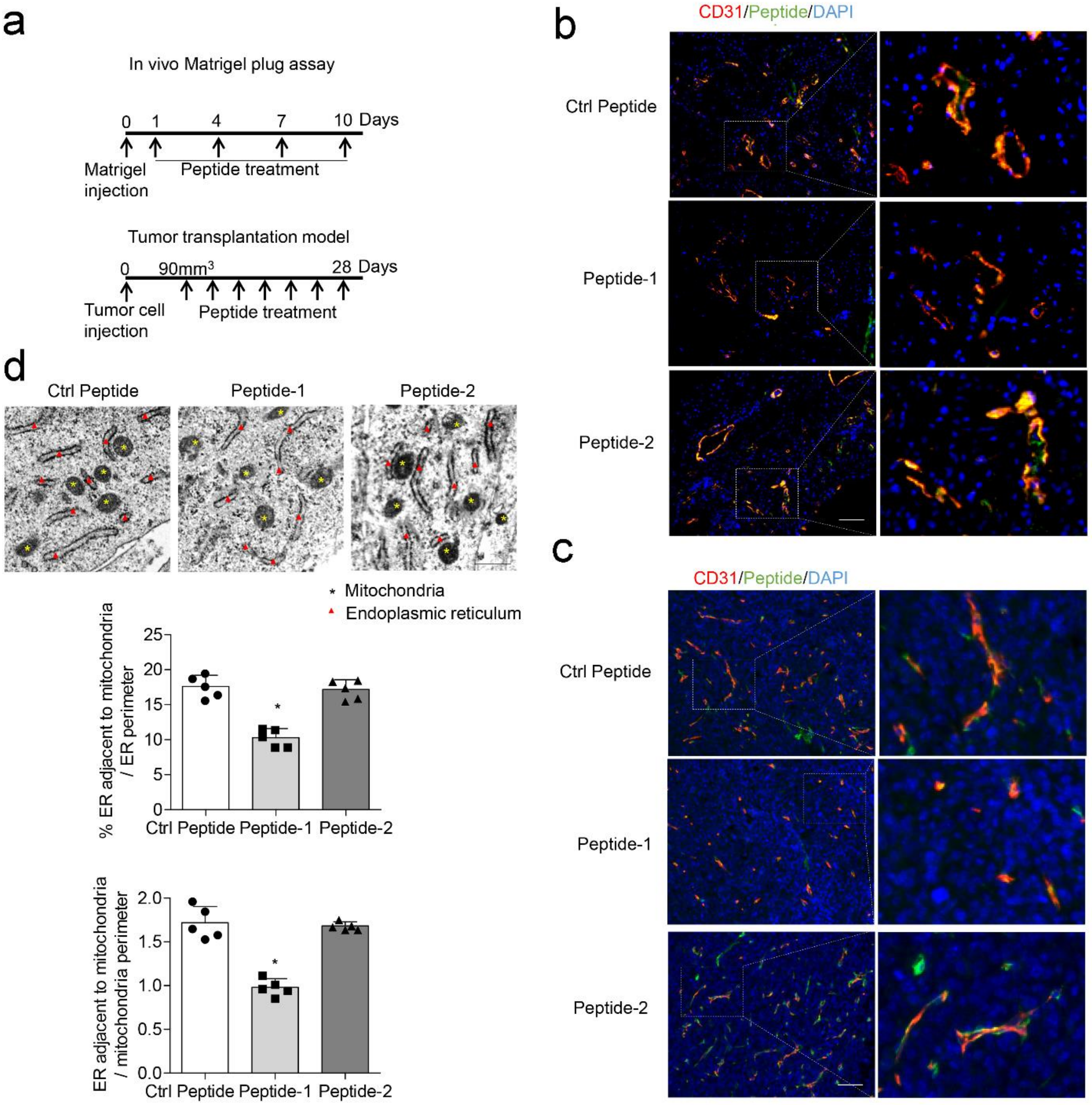

Supplementary Figure 10. (**a**) Diagram for the peptide treatment for in vivo matrigel plug assay and tumor transplantation assay. (**b**) CD31 and peptides immunostaining of matrigel plugs implanted into *FUNDC1*$^{f/Y}$*Cdh5*$^{-}$ and *FUNDC1*$^{f/Y}$*Cdh5*$^{+}$ mice for 10 days. (*n*=5 mice/group) Scale bar, 100 µm. (**c**) Representative images showing CD31 and peptides immunostaining in LLC xenografts on day 28. (*n*=5 mice/group) Scale bar, 100 µm. (**d**) Representative transmission electron microscopy images of ER and mitochondrial morphology from CD31-positive endothelial cells. Bar graph illustrating quantitation of ER length adjacent to mitochondria normalized by total ER length and by

mitochondrial perimeter (*n*=5 mice/group). Statistical significance was assessed using one-way ANOVA with post hoc multiple comparisons test for multiple comparison and presented as follows: *$p < 0.05$. All values are means ± S.D.

Supplementary Table 1. Specific primers for quantitative RT-PCR

Gene name	Forward primer sequence(5'-3')	Reverse primer sequence(5'-3')
KDR (VEGFR2) *Mus musculus*	TTTGGCAAATACAACCCTTC AGA	GCAGAAGATACTGTCACC ACC
KDR (VEGFR2) *Homo sapiens*	GGCCCAATAATCAGAGTGGC A	CCAGTGTCATTTCCGATCAC TTT
18S	GTCTGTGATGCCCTTAGATG	AGCTTATGACCCGCACTTAC
