# Supplementary material for: FUNDC1-dependent mitochondria-associated endoplasmic reticulum membranes are involved in angiogenesis and neoangiogenesis
Source: Nat Commun. 2021 May 10;12:2616. doi: 10.1038/s41467-021-22771-3 (PMC8110587; doi:10.1038/s41467-021-22771-3)
Supplement: Supplementary file 2 — Reporting Summary [file 41467_2021_22771_MOESM2_ESM.pdf]

nature research

Corresponding author(s): Cheng Wang

Last updated by author(s): Jan 16, 2021

Reporting Summary

Nature Research wishes to improve the reproducibility of the work that we publish. This form provides structure for consistency and transparency in reporting. For further information on Nature Research policies, see our Editorial Policies and the Editorial Policy Checklist.

Statistics

For all statistical analyses, confirm that the following items are present in the figure legend, table legend, main text, or Methods section.

n/a	Confirmed	
☐	☒	The exact sample size (*n*) for each experimental group/condition, given as a discrete number and unit of measurement
☐	☒	A statement on whether measurements were taken from distinct samples or whether the same sample was measured repeatedly
☐	☒	The statistical test(s) used AND whether they are one- or two-sided<br>*Only common tests should be described solely by name; describe more complex techniques in the Methods section.*
☒	☐	A description of all covariates tested
☐	☒	A description of any assumptions or corrections, such as tests of normality and adjustment for multiple comparisons
☐	☒	A full description of the statistical parameters including central tendency (e.g. means) or other basic estimates (e.g. regression coefficient) AND variation (e.g. standard deviation) or associated estimates of uncertainty (e.g. confidence intervals)
☐	☒	For null hypothesis testing, the test statistic (e.g. *F*, *t*, *r*) with confidence intervals, effect sizes, degrees of freedom and *P* value noted<br>*Give P values as exact values whenever suitable.*
☒	☐	For Bayesian analysis, information on the choice of priors and Markov chain Monte Carlo settings
☒	☐	For hierarchical and complex designs, identification of the appropriate level for tests and full reporting of outcomes
☐	☒	Estimates of effect sizes (e.g. Cohen's *d*, Pearson's *r*), indicating how they were calculated

Our web collection on statistics for biologists contains articles on many of the points above.

Software and code

Policy information about availability of computer code

Data collection	CFX96 Touch Real-Time PCR Detection System, cellSens Dimension, ZEISS ZEN Microscope system.
Data analysis	Microsoft Excel 2019, ImageJ (JACoP v.2.0), SPSS software (version 22.0, SPSS Inc), Adobe Photoshop CC2015, Microsoft Powerpoint 2019, Prism 6.0 software.

For manuscripts utilizing custom algorithms or software that are central to the research but not yet described in published literature, software must be made available to editors and reviewers. We strongly encourage code deposition in a community repository (e.g. GitHub). See the Nature Research guidelines for submitting code & software for further information.

Data

Policy information about availability of data

All manuscripts must include a data availability statement. This statement should provide the following information, where applicable:

- Accession codes, unique identifiers, or web links for publicly available datasets
- A list of figures that have associated raw data
- A description of any restrictions on data availability

The data that support the findings of this study are available within the article and its Supplementary Information or from the corresponding authors on reasonable request. Source data are provided with this paper.

Field-specific reporting

Please select the one below that is the best fit for your research. If you are not sure, read the appropriate sections before making your selection.

☒ Life sciences ☐ Behavioural & social sciences ☐ Ecological, evolutionary & environmental sciences

For a reference copy of the document with all sections, see nature.com/documents/nr-reporting-summary-flat.pdf

Life sciences study design

All studies must disclose on these points even when the disclosure is negative.

Sample size	The sample size for each experiment, n, is included in the results section and the associated figure legend.Sample size was determined to be adequate based on the magnitude and consistency of measurable differences between groups.
Data exclusions	No data were excluded from the analyses.
Replication	At least three different biological replicates has been performed for each experiment. Most of biological replicates have been performed with two or more technical replicas. Conclusion were made only when all the biological replicates were consistent. All attempts at replication were successful.
Randomization	Mice were random allocated into experimental group
Blinding	The investigators were blinded to group allocation during data collection and analysis.

Reporting for specific materials, systems and methods

We require information from authors about some types of materials, experimental systems and methods used in many studies. Here, indicate whether each material, system or method listed is relevant to your study. If you are not sure if a list item applies to your research, read the appropriate section before selecting a response.

Materials & experimental systems

n/a	Involved in the study	
☐	☒	Antibodies
☐	☒	Eukaryotic cell lines
☒	☐	Palaeontology and archaeology
☐	☒	Animals and other organisms
☒	☐	Human research participants
☒	☐	Clinical data
☒	☐	Dual use research of concern

Methods

n/a	Involved in the study	
☒	☐	ChIP-seq
☒	☐	Flow cytometry
☒	☐	MRI-based neuroimaging

Antibodies

Antibodies used	Antibodies against the following proteins were used as the primary antibodies: CD31 (BDPharmingen, San Jose, CA; 550274, 1:100 Cell Signaling Technology, Danvers, MA;77699, 1:100); β-actin and GAPDH (Santa Cruz Biotechnology, Dallas, Texas; sc-47778 and sc-137179, 1:1000); PCNA, Calreticulin, Cyt C, VDAC1, mitofusin-2 (MFN2), SRF, phosphorylated (p) SRF, VEGFR1, VEGFR2, pVEGFR2, and VEGFR3 (Cell Signaling Technology, Danvers, MA; 13110, 12238, 4280, 4661, 9482, 2893, 5147,4261, 3408, 2478, 2479 ; All dilution is 1:1000); IP3R1 (Abcam, Cambridge, MA; ab5804;1:500; ThermoFisher, Waltham, MA, PA1-901); calnexin (Abcam, Cambridge, MA; ab22595;1:500); FUNDC1 (Aviva Systems Biology,San Diego, CA; ARP53281;1:500). FUNDC1 (Novus Biologicals, LLC, Littleton CO, NBP1-81063,1:500; EMD Millipore Corporation, Temecula, CA, ABC506;1:500)
Validation	All antibodies are commercial available and validated. CD31:https://www.bdbiosciences.com/us/applications/research/stem-cell-research/cancer-research/mouse/purified-rat-anti-mouse-cd31-mec-133/p/550274;https://www.cellsignal.com/products/primary-antibodies/cd31-pecam-1-d8v9e-xp-rabbit-mab/77699; β-actin:https://www.scbt.com/zh/p/beta-actin-antibody-c4; GAPDH:https://www.scbt.com/p/gapdh-antibody-a-3;PCNA:https://www.cellsignal.com/products/primary-antibodies/pcna-d3h8p-xp-rabbit-mab/13110; Calreticulin:https://www.cellsignal.com/products/primary-antibodies/calreticulin-d3e6-xp-rabbit-mab/12238; Cyt C1: https://www.cellsignal.com/products/primary-antibodies/cytochrome-c-136f3-rabbit-mab/4280; VDAC1:https://www.cellsignal.com/products/primary-antibodies/vdac-d73d12-rabbit-mab/4661; MFN2: https://www.cellsignal.com/products/primary-antibodies/mitofusin-2-d2d10-rabbit-mab/9482; VEGFR1: https://www.cellsignal.com/products/primary-antibodies/vegf-receptor-1-antibody/2893; SRF: https://www.cellsignal.com/products/primary-antibodies/srf-d71a9-xp-rabbit-mab/5147; pSRF:https://www.cellsignal.com/products/primary-antibodies/phospho-srf-ser103-antibody/4261; pVEGFR2:https://www.cellsignal.com/products/primary-antibodies/phospho-vegf-receptor-2-tyr1175-19a10-rabbit-mab/2478; VEGFR2:https://www.cellsignal.com/products/primary-antibodies/vegf-receptor-2-55b11-rabbit-mab/2479; VEGFR3:https://www.cellsignal.com/products/primary-antibodies/vegf-receptor-3-c82a2-rabbit-mab/3408; IP3R1:https://www.abcam.com/ip3-receptor-antibody-ab5804.html; IP3R1:https://www.thermofisher.com/cn/zh/antibody/product/IP3-Receptor-1-Antibody-Polyclonal/

PA1-901;calnexin:https://www.abcam.com/calnexin-antibody-er-marker-ab22595.html;FUNDC1:https://www.avivasysbio.com/fundc1-antibody-middle-region-arp53281-p050.html;FUNDC1:https://www.novusbio.com/products/fundc1-antibody_nbp1-81063;FUNDC1:https://www.merckmillipore.com/HK/en/product/Anti-FUNDC1-Antibody,MM_NF-ABC506?ReferrerURL=https%3A%2F%2Fwww.google.com%2F;

Eukaryotic cell lines

Policy information about cell lines

Cell line source(s)	Lewis lung carcinoma (LLC) cells from ATCC, LL/2 (LLC1) (ATCC® CRL-1642™)
Authentication	Cells were amplified and frozen upon receipt from the company before their use
Mycoplasma contamination	Mycoplasma contamination has never been tested since no aberrant behavior were detected
Commonly misidentified lines (See ICLAC register)	No commonly misidentified cells lines were used in this study.

Animals and other organisms

Policy information about studies involving animals; ARRIVE guidelines recommended for reporting animal research

Laboratory animals	The FUNDC1 gene was located in Chromosome X. The CreCdh5+/- mice were purchased from Jackson Laboratory (Bar Harbor, ME). Littermate male FUNDC1f/YCdh5- mice were considered as control mice. The experimental mice were male and were used at 8-12 weeks. All mice (male) were C57BL/6J background and housed in temperature-controlled cages with a 12-h light-dark cycle, temperatures of 68-75°F with 30-70% humidity and given free access to water and food. All experimental procedures were approved by the Institutional Animal Care and Use Committee.
Wild animals	This study dit not involve wild animals.
Field-collected samples	No field collected samples were used in the study.
Ethics oversight	The animal studies were performed in compliance with the ethical guidelines for animal studies and approved by the Institutional Animal Care and Ethics Committee of Union hospital,Tongji Medical College, Huazhong University of Science and Technology

Note that full information on the approval of the study protocol must also be provided in the manuscript.
